# Prehospital Emergency Cricothyrotomy in Dogs Part 1: Experiences With Commercial Cricothyrotomy Kits

**DOI:** 10.3389/fvets.2021.705695

**Published:** 2021-09-16

**Authors:** Sureiyan Hardjo, Lee Palmer, Mark David Haworth

**Affiliations:** ^1^UQ VETS, School of Veterinary Science, The University of Queensland, Gatton, QLD, Australia; ^2^Veterinary Medical Director, National Association of Veterinary Emergency Medical Services, Auburn, AL, United States

**Keywords:** military working dogs, operational K9, CICO, intubation, cricothyrotomy, melker, portex, difficult airway

## Abstract

The surgical cricothyrotomy (CTT) has been recommended for emergency front of neck airway access (eFONA) during a cannot intubate, cannot oxygenate scenario for military working dogs (MWD) and civilian law enforcement working dogs (operational K9s). In prehospital and austere environments, combat medics and emergency medical service providers are expected to administer emergency medical care to working dogs and may only have emergency airway kits designed for humans at their disposal. The objective of this article is to provide a detailed description of the application of such devices in cadaver dogs and highlight potential alterations to manufacturer guidelines required for successful tube placement. The kits evaluated included the Portex® PCK, Melker universal cricothyrotomy kit and H&H® emergency cricothyrotomy kit. A novel technique for awake cricothyrotomy in the dog is also described, which can also be considered for in-hospital use, together with the open surgical method described for the H&H® kit. To the authors' knowledge, this is the first publication documenting and providing instruction on the application of commercial cricothyrotomy kits in dogs.

## Introduction

Military working dogs and Operational K9s serve as invaluable assets to armed forces and law enforcement agencies around the world, with their unique abilities remaining unmatched by man or machine (1).

It is accepted that primary care of critically injured dogs on the battlefield is administered by deployed human healthcare providers (2, 3) and there are formal reports of military medics rendering prehospital care to MWDs (4, 5). Due to their invaluable contribution to military operations, the money invested in their training and the strong bond with their handlers, the expectation by the U.S. military is that MWDs receive the same level of care as their human counterparts (2).

The battlefield is inherently dangerous and MWDs are likely exposed to the same risk of trauma or death as their human counterparts (4–7). When injured in combat, there is tremendous motivation to return these elite canine operators back to duty as soon as practically feasible. In order to accomplish this goal, the military continues to devote a significant degree of resources and training towards enhancing MWD combat casualty care (8). Baker et al., reported that all MWDs who survived gunshot wounds returned to duty, even those with relatively high injury scores (4). In human combat casualties, massive extremity haemorrhage, upper airway obstructions and tension pneumothorax account for the three leading causes of potentially survivable battlefield deaths (9). The most prevalent survivable combat-related deaths for MWDs currently remains unknown; however, based upon their risk for experiencing similar battlefield trauma as compared to human combat casualties, upper airway obstructions remain a potentially, survivable cause of death for the MWD as well.

Best practice recommendations for prehospital veterinary care of dogs and cats highlight that achieving airway patency and facilitating breathing are major components of the primary assessment and treatment (10). Traditionally, most references listed the tube tracheostomy (TT) as the only advised procedure for emergency front of neck airway access (eFONA) in MWDs (2), however, recommendations published within the last few years now include the CTT as a viable prehospital eFONA option for dogs (11). A recent veterinary study concluded CTT had a success rate of 100% and was faster and easier to perform when compared to TT in cadaver dogs (12). Although the requirement for a CTT in the prehospital, battlefield setting is relatively rare in people, it can be an immediately life-saving intervention (13–15). The procedure has a high success rate (13, 14, 16) and has outcomes equivalent to other methods of airway management in the prehospital setting (15).

In humans, several different techniques and commercial kits are available for performing surgical CTT in the prehospital setting (16–21). Military trauma centres and higher role field hospitals similarly utilise the surgical CTT as the preferred eFONA technique for human combat casualties (22). These medical facilities often possess a larger variety of kits, access to more medical expertise and a larger capability for performing various surgical CTT techniques as compared to the combat or field medic. Both the limited space within a combat medic's pack and the need to minimise the overall weight of the pack they must carry, constitute major limitations for the resources a combat medic has to perform a CTT. In lieu of those limitations, many light-weight, low-profile commercial CTT kits have become available on the market (23) that easily fit within most field medic packs; therefore, enhancing the combat medic's capability for performing a successful prehospital CTT. Schauer et al. inventoried 44 medic bags obtained from U.S. Army combat medics stationed at Joint-Base Lewis McChord. Their results showed that 64% carried a CTT kit with the H&H® cricothyrotomy kit as the most commonly (38%) stocked commercial kit (21). The Australian military recommends a bougie-assisted surgical CTT technique; however, the commercial Portex® cricothyrotomy kit (Smiths Medical) and the Melker universal cricothyrotomy kit (Cook® Medical) are also available.

Due to the lack of availability or access to veterinary personnel in many deployed environments and most pre-hospital settings, human healthcare providers (HCP) are expected to provide emergency care to MWDs and operational K9s suffering serious injuries (2, 3, 24). As such, it is vitally important to ensure HCPs recognise the surgical CTT as a viable eFONA technique in dogs. Additionally, they should become familiar with the differences in techniques from CTTs in humans and how to apply their kits when performing CTTs in dogs. Human healthcare providers should refer to their jurisdictional laws regarding the delivery of treatment to operational K9s by non-veterinarians. Nine states in the U.S. and seven of eight states and territories in Australia formally allow basic emergency care or transport of animals by non-veterinarians. However, provision of care, including advanced airway management may not be lawful in other states or countries.

Specific indications and contraindications for CTT in dogs have been described elsewhere (10, 11). In general, orotracheal or endotracheal intubation remains the recommended first-line airway management technique for establishing a definitive airway in dogs (2, 10, 11, 24, 25). Cricothyrotomy should only be performed if orotracheal intubation fails of cannot be performed for any reason, often described as cannot intubate, cannot oxygenate (CICO).

This report assesses three commercially available CTT kits that have the potential to be used in the pre-hospital or field hospital setting. We discuss the practicality of preparing for airway management and present detailed methodology on applying these kits on cadaver dogs based our experiences. A method for an awake CTT is also presented using basic equipment and instruments, which together with the surgical method, can also be considered for in-hospital use by veterinarians during a CICO emergency.

## Materials and Equipment

An ethics certificate was granted from the University of Queensland Production and Companion Animal Ethics Committee, certificate number ANRFA/159/20 for use of 10 cadaver dogs. Cadaver dogs were reused from unrelated teaching practicals. The population of dog cadavers consisted of two Greyhounds six Mastiff crosses, a Bull terrier cross and a Rottweiler. Body weights were normally distributed averaging 28.6 kg with a standard deviation of 4.39 kg. All CTTs were performed by SH, who has prior experience in performing the procedure.

### Cricothyrotomy Kits

**Figure 1:**
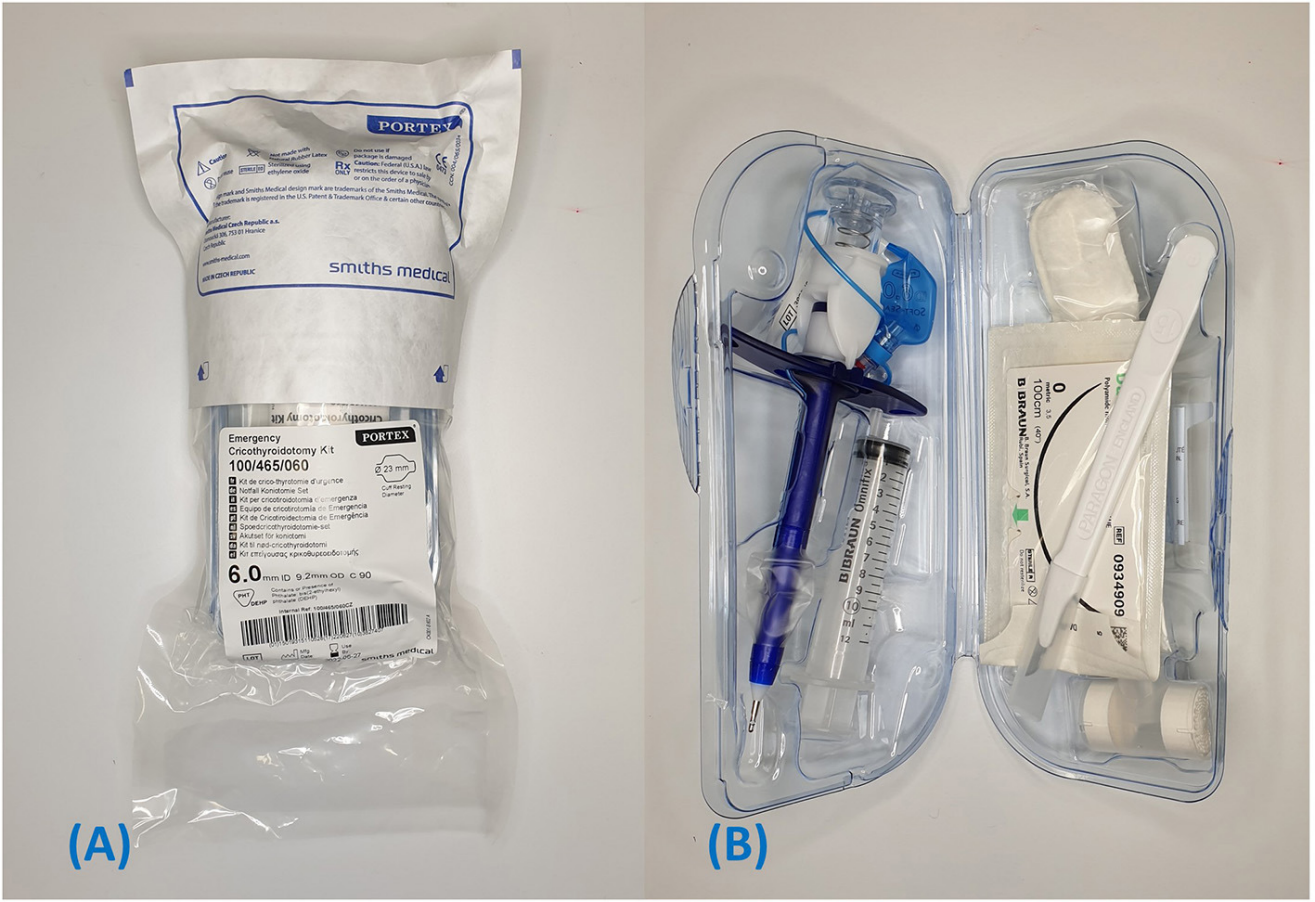
Portex**®** PCK. **(A)** Package when removed from the box. **(B)** Contents of inner container.

**Figure 2:**
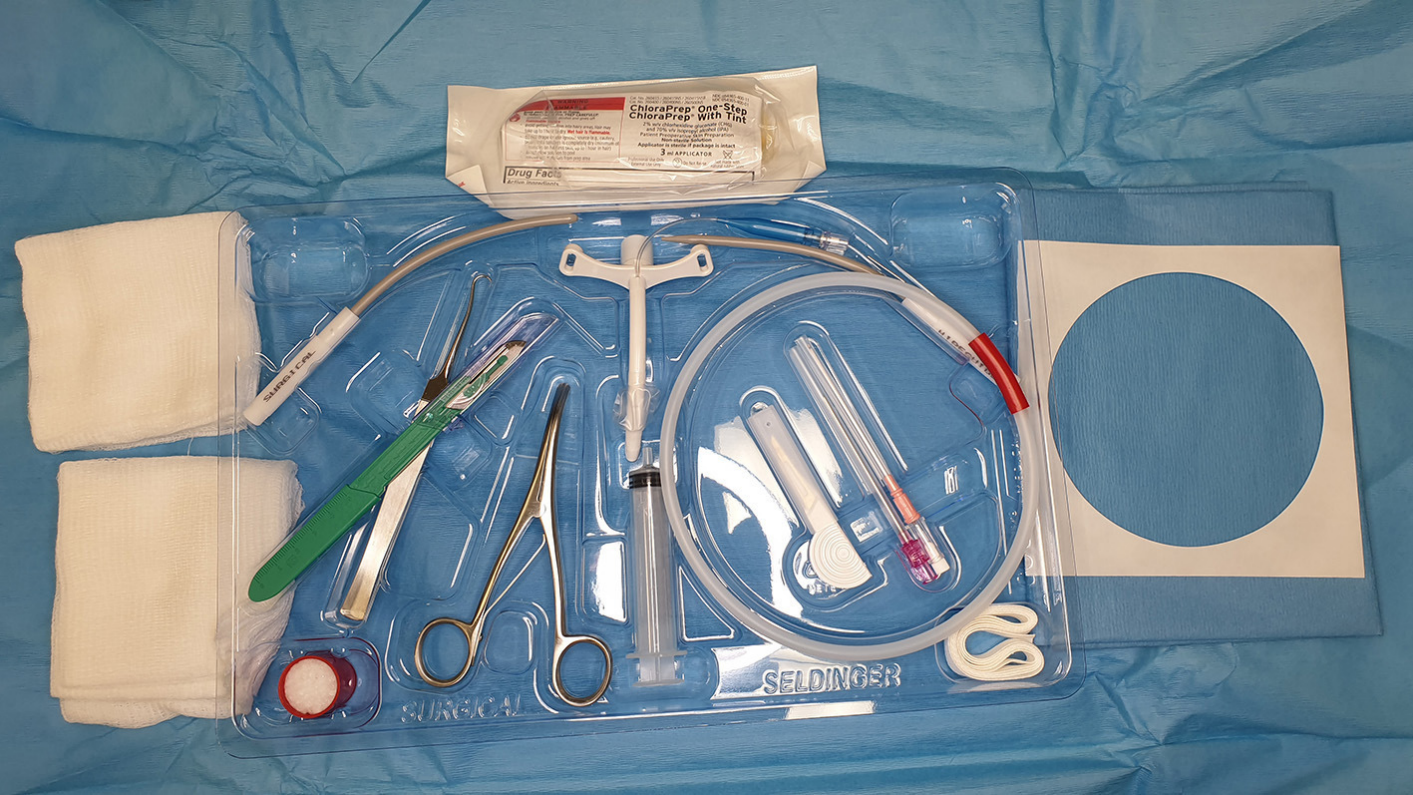
Melker universal cricothyrotomy kit.

**Figure 3:**
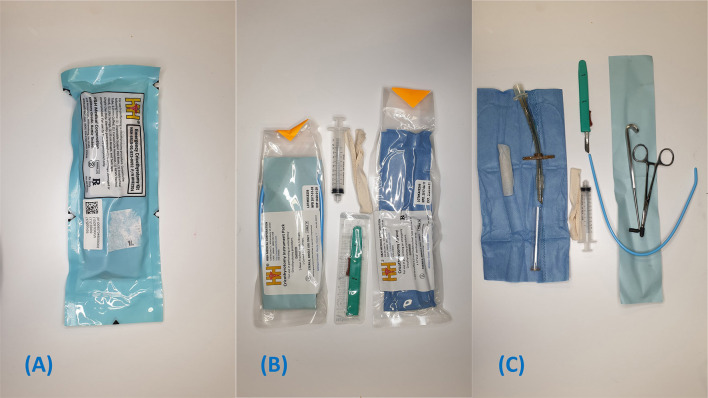
H&H emergency cricothyrotomy kit and its contents. **(A)** Original package. **(B)** Contents of H&H kit after outer package is removed. **(C)** Instruments included in the H&H Kit.

Cricothyrotomy kits were obtained based on availability and to represent three different styles of emergency CTT kits that can be used in the prehospital environment;

1. Portex® cricothyrotomy kit (PCK) (Smiths Medical)^1^: Bespoke all-in-one device (Figure 1)
2. Melker universal cricothyrotomy kit (Cook® Medical)^2^: Seldinger technique (Figure 2)
3. H&H® cricothyrotomy kit (H&H Medical Corporation®)^3^: Open, surgical technique (Figure 3).

These kits were applied to cadaver dogs based on the manufacturer's recommendations. Images and video were recorded of tube insertions. If complications were encountered during insertion of devices, steps were modified to facilitate a successful procedure as described in the methods section below.

Other materials and equipment used included:

- A 2.6 mm external diameter, polypropylene rigid dog urinary catheter
- An unbranded 8.0 mm internal diameter, 10.0 mm external diameter, cuffed endotracheal tube
- A size #22 and #10 scalpel blade.

A video endoscope was used to capture endoscopic photos and videos. The details of this equipment are as follows:

- Olympus Xenon Exera II CV −180 Video processor with keyboard & scope cable
- Olympus Xenon Exera II CLV −180 Light source & water bottle
- Olympus gastroscope GIF Q160. Length 1,345 mm, working length 1,030 mm, scope tip diameter 9.5 mm, biopsy channel 2.8 mm, angulation 4 way.

## Methods

### Preparation

Successful achievement of eFONA warrants administration of sedation and/or anaesthesia. Rapid sequence induction (RSI) is a common technique designed for quickly achieving an adequate depth of anaesthesia to manage a patient with a compromised airway. A canine RSI protocol has been described previously (11) and consists of a combination of ketamine, a full-mu opioid agonist (fentanyl, hydromorphone) and a benzodiazepine (midazolam). Although paralytics (neuromuscular blocking agents) are frequently used in human RSI protocols, they are not routinely recommended or warranted in dogs. Other agents such as dexmedetomidine and propofol are also commonly utilised in veterinary medicine. Considering that eFONA is performed as a last resort following a failed intubation attempt, expect that many patients have already received some degree of anaesthesia prior to making the decision to perform a CTT; therefore, reducing the amount of systemic anaesthetics required for completing the procedure. Subcutaneous infiltration of a local anaesthetic (lignocaine or bupivacaine) at the intended surgical site may further reduce the amount of systemic anaesthesia required. Under an extreme urgent situation involving a conscious patient, an alternative approach may consist of only performing subcutaneous infiltration of local anaesthetics at the intended surgical site without administering any other systemic anaesthetic agents (e.g., ketamine); otherwise known as an “awake” CTT (17). After the airway is secured, longer acting, systemic analgesics should be administered, such as hydromorphone or morphine, whilst en route to a medical facility.

### Patient Positioning

Ideally, the patient is positioned in dorsal recumbency (supine) unless attempting an awake cricothyrotomy. A towel, piece of clothing or an intravenous fluid bag may be placed below the dorsal cervical region to hyperextend the neck (11). This pushes the laryngeal structures superficially, allowing easier localisation of surface landmarks.

### Laryngeal Handshake

The laryngeal handshake is the technique used to identify surface landmarks for tube CTT. This is particularly important with percutaneous techniques or limited surgical approaches. It is the opinion of the authors that the surface landmarks in the dogs are far more prominent and easier to identify than in people. McCarthy et al. observed a lower success rate when non-veterinary medical professionals performed CTT in dog cadavers as compared with human cadavers (26). To account for anatomical differences between species, the authors recommend that HCPs become familiar with the landmarks and performance of the technique before attempting the CTT in a dog. A previous study validated the effectiveness of the laryngeal handshake technique in dogs. In that study, novice veterinary students achieved 100% success rate in placing tube CTTs in cadaver dogs when using the laryngeal handshake technique (12). The technique described in the aforementioned study is as follows:

1. With the patient in dorsal recumbency, the operator is positioned on the patient's right if right hand dominant and the patient's left if left hand dominant.
2. Palpate the trachea on midline on the caudoventral cervical region;
3. Run the index finger of the nondominant hand cranially until the first firm structure is palpated (this is the ventral cricoid cartilage);
4. Palpate the cricothyroid membrane (CTM) as the index finger drops into a soft tissue depression immediately cranial to the cricoid cartilage;
5. Palpation of the CTM with the index finger continues while the thumb and remaining fingers grasp the thyroid cartilage to stabilise the larynx;
6. Shift the thumb and index finger caudally to tension the skin for incision over the CTM; and
7. The location of the CTM can be rechecked if necessary during the procedure by repeatedly running the index finger over the cricoid cartilage in a caudo-cranial direction.

A video of the surface landmarks and laryngeal handshake can be found in the footnotes^4^.

### Skin Incision

Although the prehospital environment does not always allow ideal conditions for surgically prepping the intended surgical site, always attempt to utilise aseptic technique when possible. The incision is centred over the cricothyroid membrane, which is located using the laryngeal handshake technique.

Prepare the surgical site by clipping a 10 x 5 cm section of hair over the ventral cervical region (neck), centred over the larynx. Remove all clipped hair and gross contaminants and perform a surgical skin-prep utilising the appropriate technique and disinfectant. The authors recognise the challenge of finding or possessing electric hair clippers in the prehospital setting. Some commercial CTT kits designed for humans come with equipped with a skin razor; however, it generally not advised to use these human-designed skin razors on dogs to avoid unnecessary trauma to the skin.

**Figure 4:**
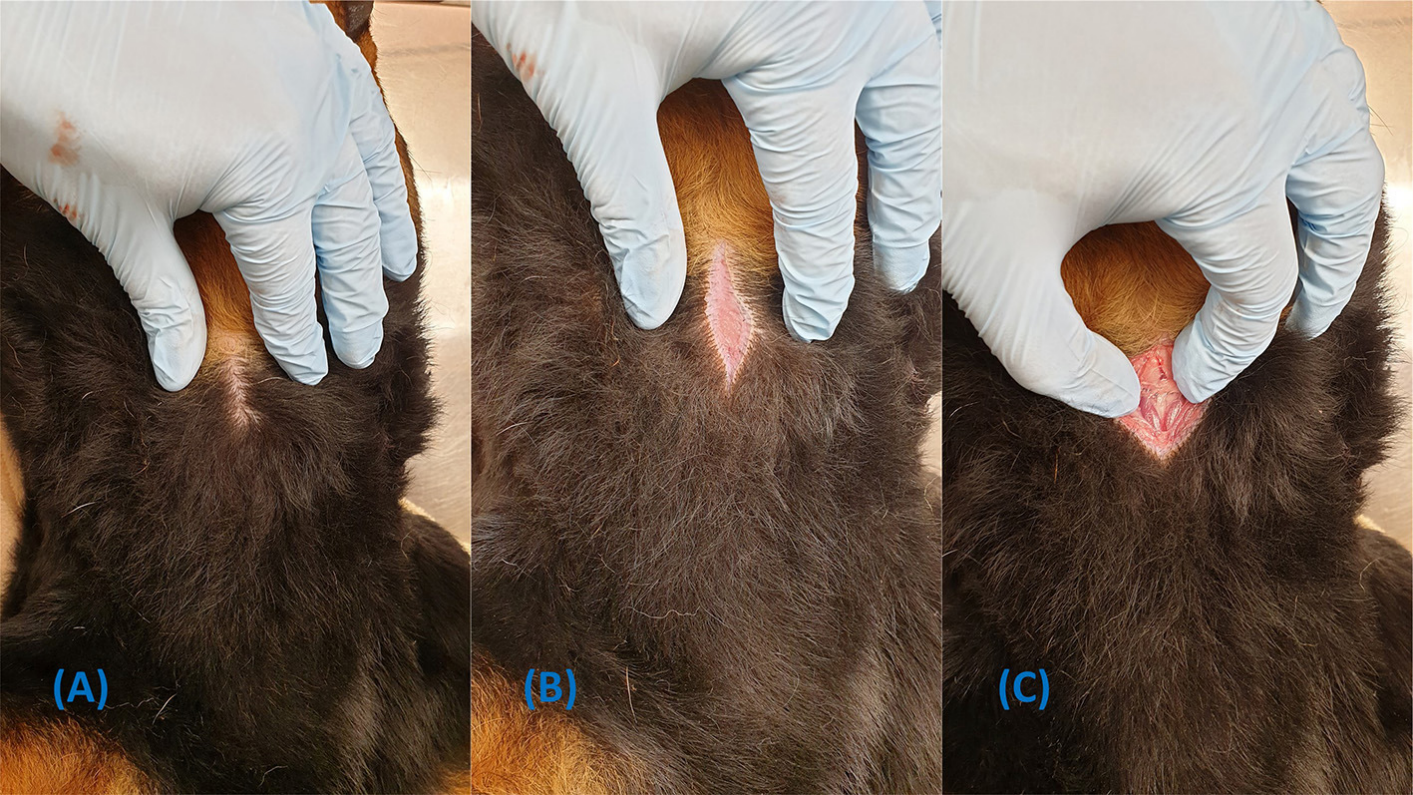
Demonstration of how to perform a skin incision without clipping the fur. **(A)** Part the fur over the intended incision site. **(B)** Appy tension while the incision is made. **(C)** Dissection continues through the skin incision.

If the skin cannot be clipped, the incision may be performed with minimal contamination by parting the fur and maintaining firm tension perpendicular to the incision (Figure 4). A skin preparation with a disinfectant or clean water should still be attempted before incising.

### Portex® PCK

The PCK was originally packaged in a cardboard box. The wrapping required removal before a plastic clamshell container could be opened (Figures 1A,B). The packaging allows sequential retrieval of equipment as required. Instruments located to the right of the kit include a #15 scalpel, suture, neck band and T-tube (a tube adapter that provides heat and moisture exchange). To the left there is the Portex® 6.0 mm internal diameter CTT tube with dilator and veress needle/indicator preloaded within the lumen (ready to use as-is) and a 12 ml syringe.

### Portex® PCK Insertion Technique

**Figure 5:**
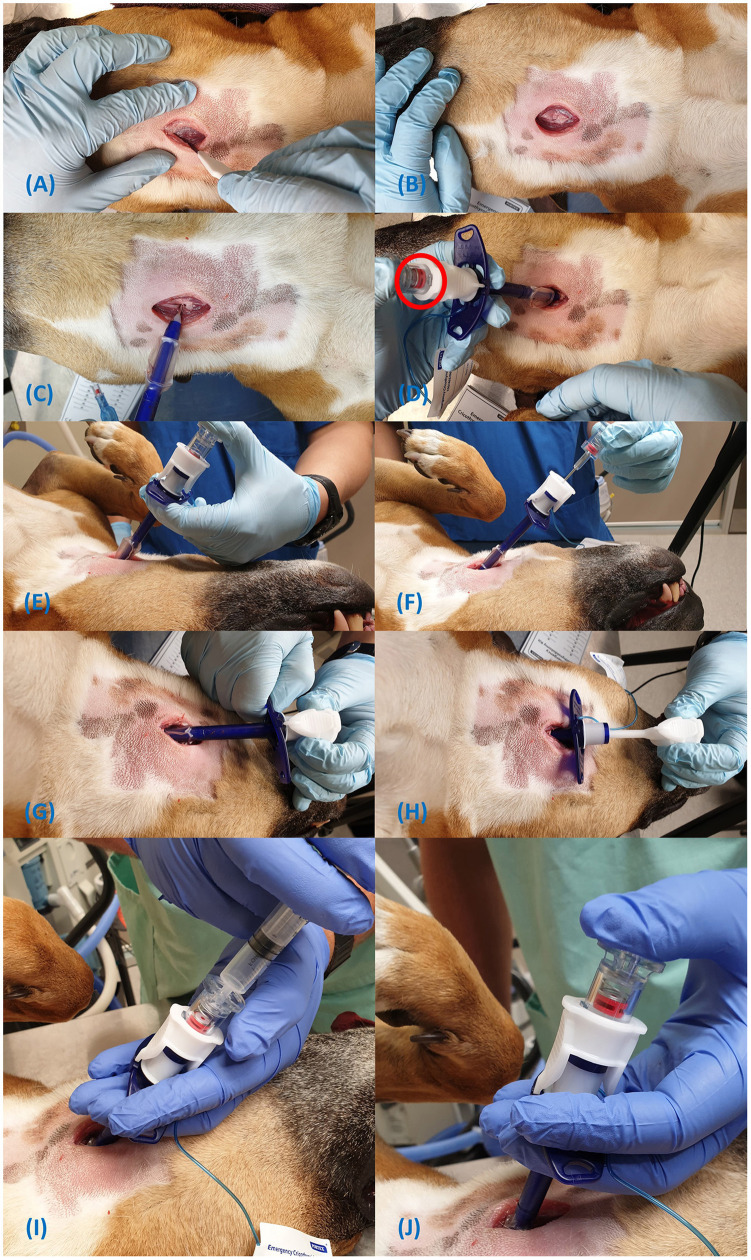
Steps for insertion of the Portex® cricothyrotomy tube **(A-J)**.

**Figure 6:**
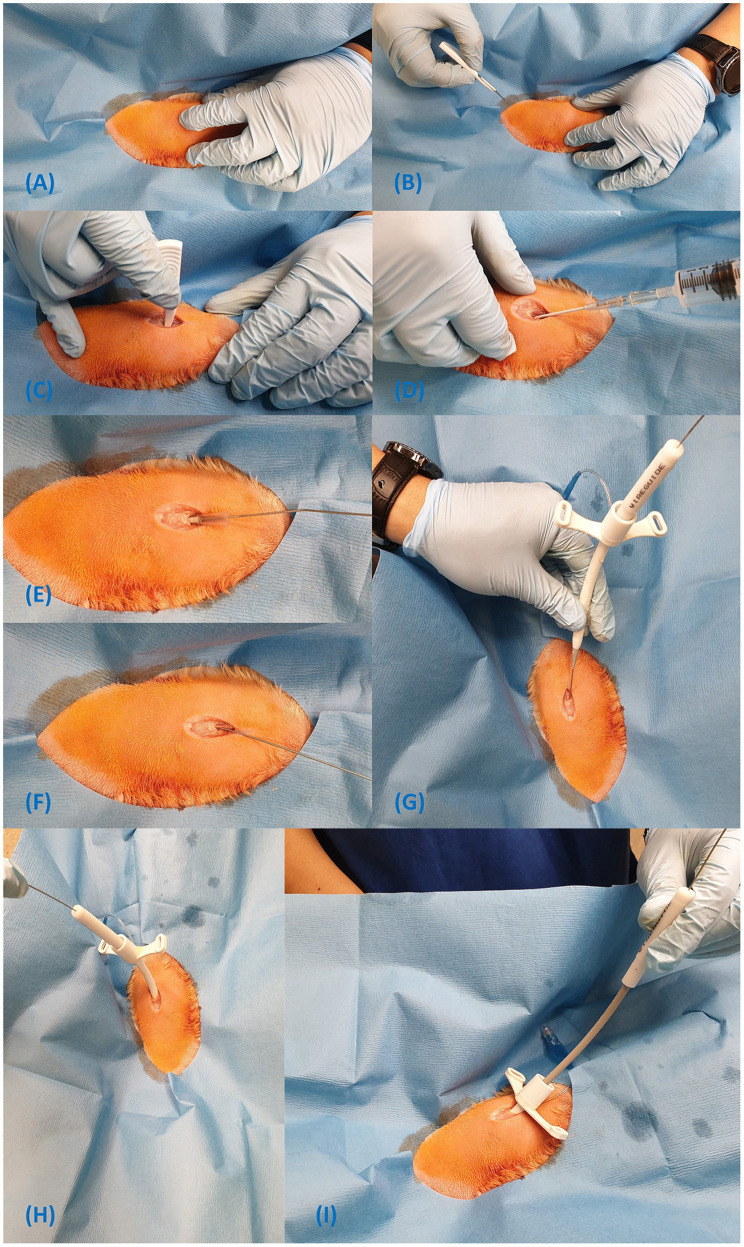
Steps for insertion of the Melker cricothyrotomy tube using the Seldinger technique **(A–I)**.

1. Locate the CTM using the laryngeal handshake technique;
2. Make a sagittal 3 cm skin incision over the CTM. However, a horizontal incision may be used as per the manufacturer's instruction (Figure 5A);
3. Dissect the sternohyoid muscle on midline to expose the ventral larynx (Figure 5B);
4. Place the metal tip of the Portex® tube/dilator assembly over the CTM and apply pressure from the top of the device in a dorsal direction, perpendicular to the CTM (Figures 5C,D). Note, the veress needle will automatically detach from the device when a certain pressure is exceeded, regardless of whether the CTM is punctured. Hence, force must be applied from the top to prevent detachment.
5. The indicator flag (Figure 5D, circled in red) will raise upon contact with the CTM and lower once it is penetrated. A click will be audible once the airway is penetrated (Figures 5D,E);
6. (Optional step) Placement in the airway may also be checked by aspirating air from the top of the device with the included syringe (Figure 5I). Once sufficiently in the airway, the indicator will raise again as the veress needle contacts the dorsal airway (Figure 5J);
7. Remove the veress needle and direct the device caudally (Figures 5F,G);
8. Push the tube caudally into the airway over the dilator and remove the dilator (Figures 5G,H);
9. Secure the tube around the neck with the provided neck band; and
10. Inflate the cuff only if positive pressure ventilation is required.

Video of PCK insertion with operation of the indicator flag can be found in the footnotes^5^. The manufacturer's procedure instructions for the PCK can be found in the Supplementary Notes.

### Melker Universal Cricothytoromy Kit

The Melker universal cricothyrotomy kit is presented in a sterile plastic tray with a peel-away paper covering adhered to the top. The kit is wrapped in a sterile pack wrap, which must be unfolded. Items immediately accessible are a ChloraPrep skin preparation device, sterile adhesive patient drape and gauze swabs. After removal of these items, all equipment required for performing the CTT is immediately accessible. The CTT tube is centrally located and the tools required for a Seldinger CTT and surgical CTT are to the right and left of the tray respectively. The author's recommendations for surgical techniques are covered in the H&H and awake CTT sections.

### Melker Cricothyrotomy Tube Insertion Using the Seldinger Technique

The Seldinger technique involves a limited approach to the CTM

1. Prepare the tube assembly by passing the seldinger dilator, located to the upper right of the try into the proximal end of the CTT tube until it stops;
2. Identify the CTM using the laryngeal handshake (Figures 6A,B);
3. Make a 1–2 cm sagittal skin incision directly over the CTM with the included #15 blade (the skin incision must be large enough to accommodate the diameter of the airway device);
4. Dissect through the muscle and puncture the CTM with the scalpel to allow smooth passage of the dilator and airway device (Figure 6C);
5. Attach the syringe to the catheter's stylet and introduce the catheter in to the CTM incision in a caudal direction at a 45-degree angle (Figure 6D);
6. Aspirate the plunger of the syringe while advancing the catheter and loss of resistance will indicate entry into the airway;
7. Feed off the catheter into the airway, removing the stylet and syringe;
8. Pass the soft end of the guidewire into the catheter for about 10 cm and remove the catheter. Do not let go of the guidewire at any point (Figures 6E,F);
9. Leaving the guidewire *in situ*, pass the dilator/tube assembly over the guidewire and grasp the proximal end of the guidewire as it emerges through the proximal end of the dilator (Figure 6G);
10. Hold the handle portion of the dilator and continue to advance the dilator/tube assembly into the airway with a twisting motion until the tube is completely in the airway (Figure 6H); Note, the handle portion must be held or the dilator will not advance.
11. Withdraw the guidewire and dilator from the lumen of the CTT tube (Figure 6I); and
12. Secure the device to the patient's neck using the included neck band, located in the bottom right corner of the pack.

Please see the footnotes for a link to a video demonstration of the Melker kit insertion technique^6^.

The manufacturer's procedure instructions for the Melker kit can be found in the Supplementary Notes.

### H&H® Emergency Cricothyrotomy Kit

The contents of the H&H® kit are depicted in Figure 3. The kit is presented as a blue plastic packet with five tear points distributed over three sides. Within the main packet, there are two smaller packs, a syringe, retractable #10 scalpel and a fabric band to secure the tube to the patient Figure 3B. The instrument packet contains curved forceps, tracheal hook, and gum elastic bougie. The tube pack contains the 6.0 mm internal diameter cuffed cricothyrotomy tube.

The H&H cricothyrotomy procedure used in this study was adapted from the original rapid four-step technique (18) and the bougie assisted CTT, first described by Smith (19).

### H&H® Cricothyrotomy Tube Insertion Using the Open Surgical Technique

**Figure 7:**
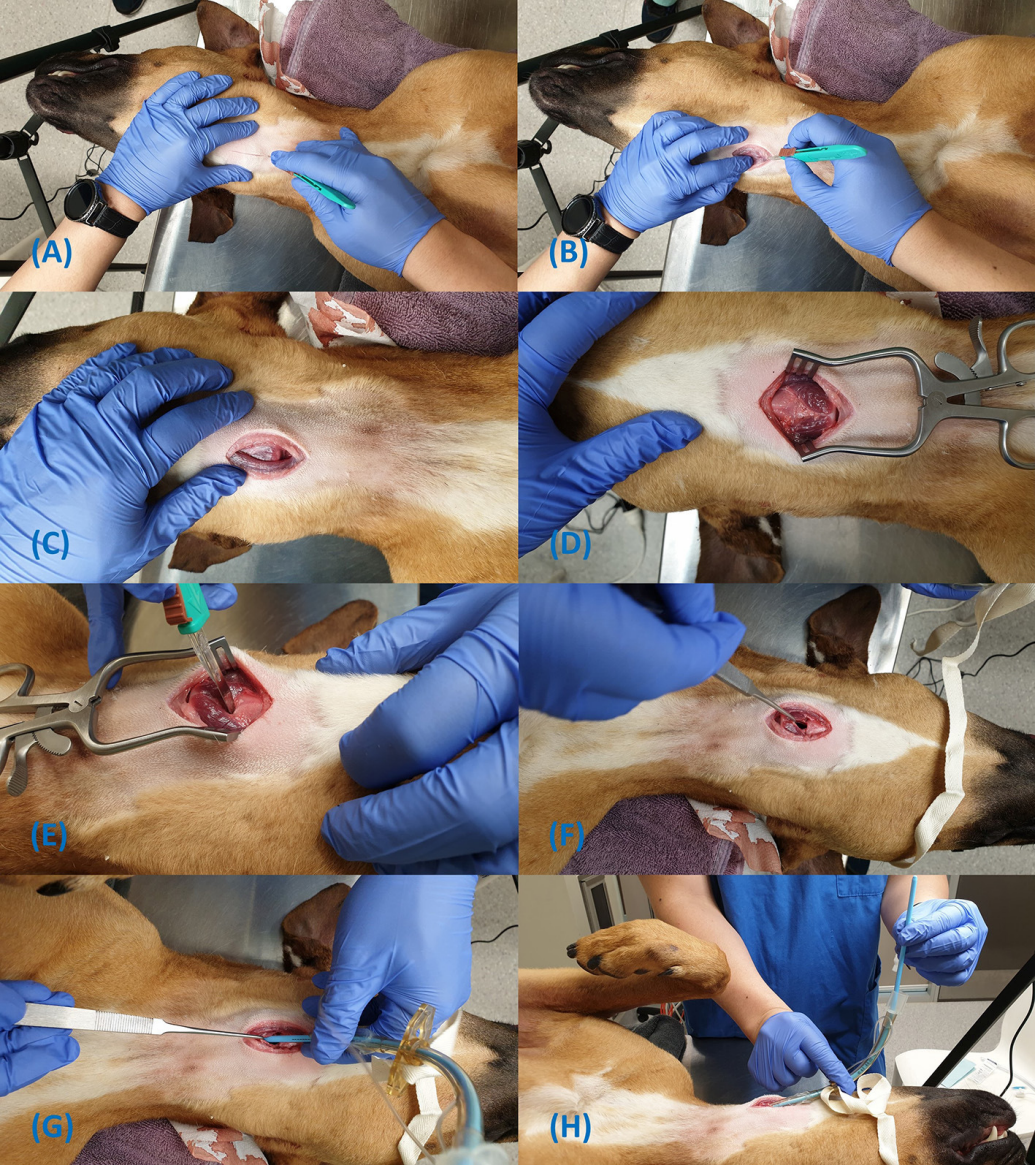
Steps for insertion of the H&H cricothyrotomy tube using the open surgical technique **(A–H)**.

1. Locate the CTM using the laryngeal handshake technique;
2. Make a 3–4 cm vertical skin incision over the CTM (Figure 7A);
3. Incise the sternohyoid muscle and bluntly dissect on midline to expose the ventral larynx (Figures 7B–D);
4. Make a stab incision through the CTM, avoiding inadvertent incision of the cricothyroideus muscle (Figure 7E);
5. Place the tracheal hook over the ventral cricoid cartilage and pull the larynx caudoventrally (Figure 7F);
6. Gently insert the bougie into the airway (Figure 7G);
7. Pass the tube over the bougie into the trachea and remove the bougie (Figure 7H);
8. Secure with the provided neck band; and
9. Inflate the cuff if the patient requires positive pressure ventilation.

### Emergency Awake Cricothyrotomy

Equipment required for the awake CTT includes a #10 or #22 scalpel, appropriately sized endotracheal tube (~half the diameter expected for endotracheal intubation), rigid dog urinary catheter or gum elastic bougie. It is possible to use any of the commercial kits above if required. This technique is based on the CTT described by Hardjo et al. (27) for use in dogs. The scalpel-finger-tube technique (20) may also be performed if a bougie is not available.

### Emergency Awake Cricothyrotomy Technique

**Figure 8:**
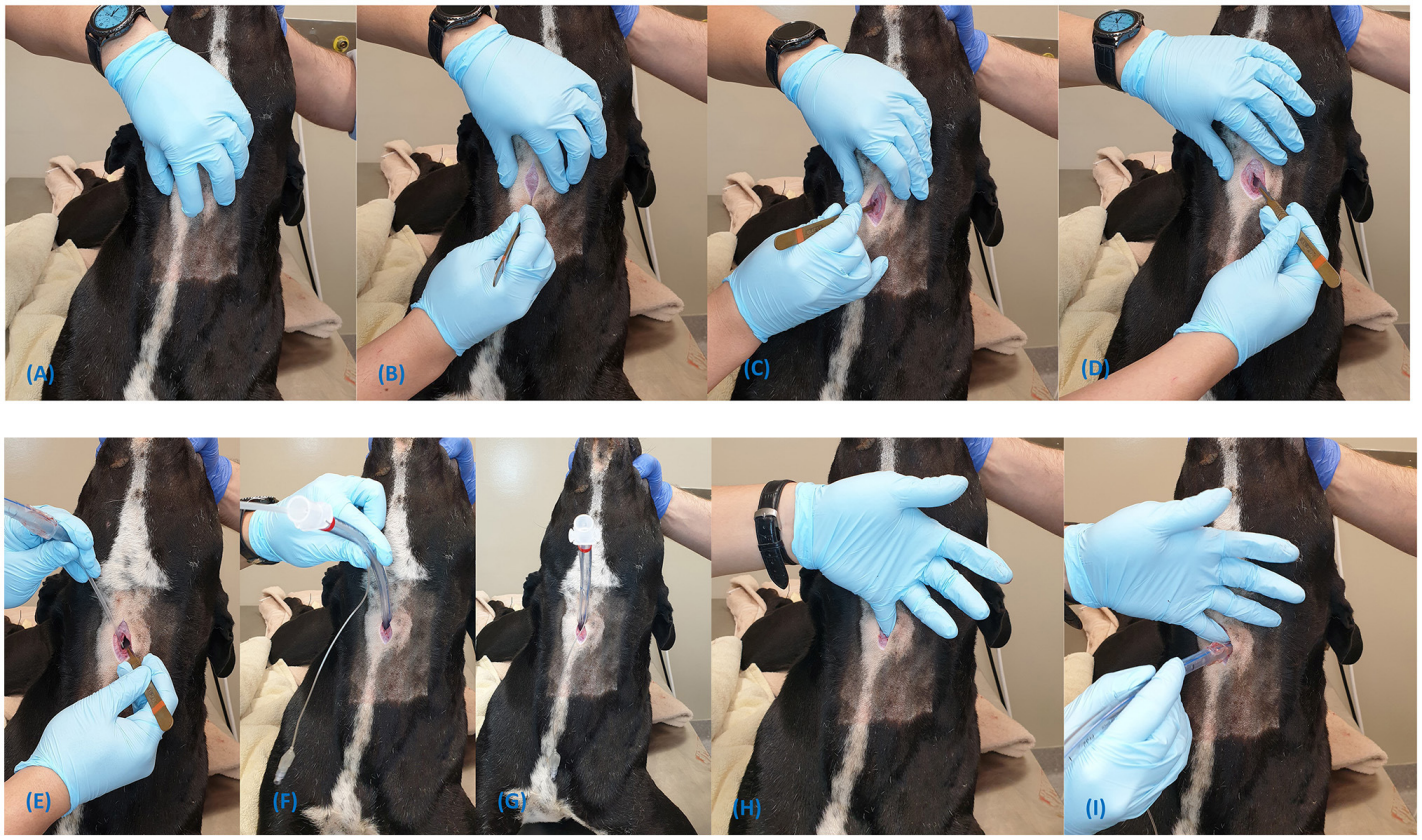
Steps for the emergency awake cricothyrotomy **(A–G)**. The scalpel-finger-tube technique is demonstrated in **(H,I)**.

1. Prepare the equipment by placing the urinary catheter bougie within the lumen of the endotracheal tube.
2. Position the patient in sternal recumbency (prone) with the neck fully extended (Figure 8A);
3. Perform the laryngeal handshake (Figure 8A);
4. Administer 2% Lidocaine (without epinephrine) subcutaneously and into the soft tissues overlying the CTM;
5. Make a 3–4 cm sagittal skin incision, centred over the CTM (Figure 8B);
6. Incise through the sternohyoid muscle on midline;
7. Make a stab incision through the CTM with the scalpel and extend as required (Figure 8C);
8. Note, the sternohyoid incision and the extension of the CTM incision is not required if using a large scalpel blade, such as a #20 or # 22.
9. Rotate the blade 180 degrees (proximally) to prevent bougie trapping and use the flat surface of the blade to retract the soft tissues laterally (Figures 8C,D);
10. Firmly grasp the urinary catheter bougie alone and pass it into the airway 10 cm distally (Figure 8E); Note: If a bougie is not available a scalpel-finger-tube technique may be used as depicted in Figures 8H,I.
11. Pass the endotracheal tube over the urinary catheter bougie into the airway whilst firmly grasping the proximal end of the urinary catheter bougie. Remove the bougie (Figures 8F,G);
12. Secure the tube around the neck using umbilical tape or bandage material; and
13. Once airway control has been achieved, longer acting analgesia should be administered.

Please see the footnotes for a link to a video demonstration of the emergency awake cricothyrotomy^7^.

## Results

### Portex®

When following the manufacturer's recommendations of performing a skin incision only, the tube and dilator assembly failed to advance into the airway. A minor muscle dissection was subsequently performed but the tube still failed to advance, even following successful puncture of the CTM by the veress needle. Sharp dissection of overlying soft tissues to the level of the CTM with the scalpel provided, was essential to allow insertion of the tube as it was designed. In practice, the level of dissection required can be easily gauged by direct palpation of laryngeal structures with no soft tissue in between.

### Melker

**Figure 9:**
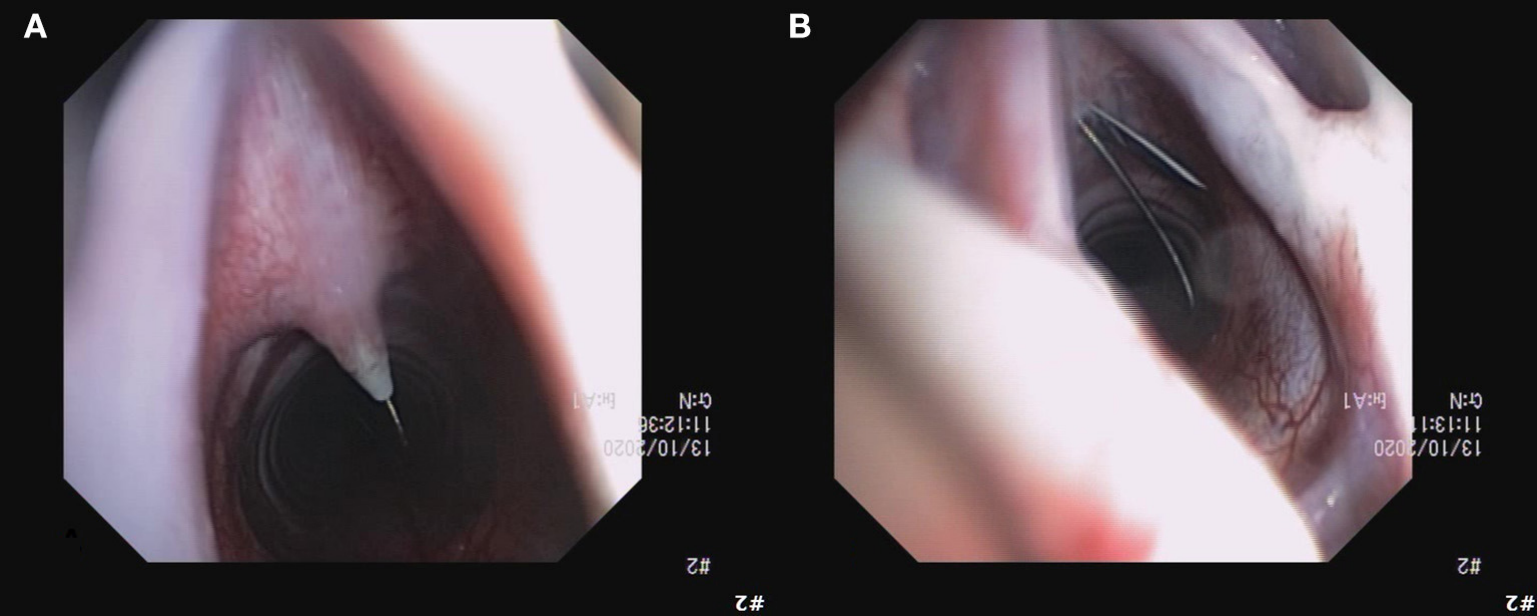
Image of unsuccessful dilation over the guidewire **(A)**, requiring a stab incision in to the CTM with the guidewire *in situ* **(B)**. Subsequent procedures with the Melker kit implementing an incision in to the CTM before placing the guidewire were successful without complications.

When applying the Melker kit using the seldinger technique, there were three levels of dissection performed. A skin incision alone was performed as per the manufacturer's instructions, and the tube/dilator assembly failed to pass between the sternohyoid muscles (Figure 9A). Further dissection was made through the muscle and it appeared the dilator could be pushed further, but the tube itself still failed to pass. Finally, an incision was made in the CTM and the tube passed smoothly (Figure 9B).

### H&H®/Surgical

The endotracheal tube in the H&H® kit had a collar adhered to the distal third of the tube to allow attachment of the neck band. This collar was not adjustable and prevented insertion of the tube into the airway beyond this point.

Although the anatomy of the ventral larynx and CTM is clear in the images (Figures 7C,D), this level of dissection is for instructive purposes only. Normally, these steps are guided with palpation of anatomical landmarks and are not always directly visualised. Hence, the incision into the CTM as pictured is not obvious and the use of the bougie during CTT is strongly recommended to guide tube insertion as it was shown to be faster than the standard surgical technique (28).

### Awake CTT

In a similar theme to the other approaches, a single stab incision with a #10 blade failed to allow passage of a large tube (8.0 mm I.D.) in one attempt. The #10 blade used in Figures 3A–D was used to demonstrate the ability to insert larger tubes (8.0 mm I.D, 10.0 mm E.D) by lengthening the muscle and CTM incisions despite having a relatively small blade. A #22 blade allowed a simple stab approach through muscle and the CTM in one motion to accomodate a 6.0 mm I.D tube, as demonstrated in the video^7^.

## Discussion

The introduction and application of CTT to canines for eFONA is yet to gain widespread awareness and withstand the rigours of veterinary practice to gain acceptance. Despite this, the CTT is the procedure of choice during CICO emergencies in human medicine (29). It has also recently been shown to allow adequate ventilation in a porcine model (27) and demonstrated faster placement times compared with TT with 100% accuracy in dogs (12). Access to equipment such as these CTT kits, and knowledge of their application, is essential for life-saving interventions for CICO situations in operational K9s and MWD in the prehospital setting. However, further investigation with clinical applications is required to fully elucidate its advantages and complications in small animals.

### Incision Orientation

A vertical incision and splitting of the muscle on midline is recommended by the authors as this allows a less traumatic approach. A sagittal incision and splitting of the muscle on midline is recommended by the authors as this allows a less traumatic approach, avoiding transecting fibres from the sternohyoideus and cricothyroideus muscles (Figure 7C). The anastomosing cranial cricothyroid artery found in some humans has not been described in the dog and vertical incisions through the CTM should be safe (Figures 6C, 8C). Furthermore, re-orientation of the blade and reestablishment of anatomical landmarks during the procedure does not need to be performed as with a horizontal incision (29).

If the CTM can be visualised, a horizontal incision as per human recommendations is also reasonable (Figure 7E). Benefits of a horizontal incision are that HCPs do not need to re-learn the technique and can somewhat rely on experience and muscle memory from previous training. If already familiar with this technique, it is reasonable to recommend prehospital operators continue using it to reduce cognitive demand in high-stress situations.

The authors found that when the cricothyroid ligament (central portion of the CTM) is transected with a horizontal incision, the tension perpendicular to the ligament readily expands the cricothyroid space, which can be beneficial for large tubes or expedient airway access in an emergency. This was not seen with vertical incisions as the central fibres remain intact and the stoma is limited to the original length of the incision. Furthermore, the anatomical function of the cricothyroid ligament remains with vertical incisions, preventing hyperextension at the cricothyroid articulation.

### Soft Tissue Dissection

As the CTM in humans lies immediately below the skin, instruction from the percutaneous kits only require skin incisions large enough to accommodate the CTT tube and no further dissection. However, in dogs, the two muscle bellies of the sternohyoid are joined on midline and completely cover the ventral larynx and CTM (30). Despite puncture into the airway with the veress needle in the Portex® and catheter in the Melker kit, the dilators were insufficient to allow tube passage through the sternohyoid muscle. Therefore, a common complication encountered by the authors with limited approaches in dogs, was failure to pass the cricothyrotomy tube through the soft tissues superficial to the CTM. This difficulty may be encountered presumably because dilators are designed to penetrate the CTM alone in humans and are unable to consistently separate soft tissue easily. In a study assessing percutaneous dilatational tracheostomy in dogs, the technique involved two dilators of increasing size to create a suitable stoma. However, despite these efforts, subcutaneous placement of the tube and tracheal ring fractures were observed complications, suggesting poor stoma formation and the requirement of excessive force to create soft tissue tracts via dilation in dogs (31). It is reasonable to consider stoma formation through the muscle layer in the dog is difficult using dilation devices and the authors recommend dissection of a suitable tract to the level of the CTM to increase the chance of success.

### Portex® vs. Melker

It is notable that the PCK and Melker both used a dilatational method to penetrate the CTM but required different levels of soft tissue dissection for successful tube placement. The PCK utilised a mechanism where a pilot hole is created in the CTM by the veress needle before introducing the dilator and the tube. In our experience, the CTM was dilated with this device alone, provided there was adequate dissection through the sternohyoid. On the other hand, the pilot hole for the Melker kit is created using the seldinger technique with a small over the needle catheter and the guidewire to direct the dilator. However, incision through the CTM was required for tube placement for the Melker, whereas it was not for the PCK. It is possible this pilot hole was too small to allow adequate separation of the cricothyroid ligament. Differences in dilator design could also reasonably explain the requirement for CTM incision for successful application of the Melker device. The Melker's dilator is curved, so a dorsally applied force may simply deform the apparatus or be redirected caudally rather than in to the CTM. The PCK device is straight and force can be more easily directed dorsally to separate the CTM. Nevertheless, once landmarks are identified and dissection commences, it takes a fraction of a second to direct the scalpel dorsally and make a stab incision through the CTM for the Melker kit. Performing this extra step is likely to improve first-time success rate for kits using tissue dilators.

### Practicality of H&H

The H&H® kit may prove practically challenging to use in a prehospital environment. There are numerous packets that require opening which further delays tube insertion, potentially prolonging hypoxaemia. Once open, it is conceivably difficult to maintain sterility of instruments as there is only a small drape provided in the tube packet to lay the instruments upon before use. Operators should consider utilising a large drape to lay instruments before use or institute the help of an assistant to open packets and pass instruments.

The collar for the neck band only allowed an ~6 cm length of tube in the airway and was not adjustable. This mechanism seemed somewhat illogical as having any length of tube proximal to this collar only adds resistance and dead space to the system. Furthermore, the short section of the tube in the airway would have a greater potential to dislodge, particularly with increased leverage of the proximal portion of the tube. A shorter tube or allowing an adjustable collar may optimise function and reduce the chance of tube dislodgement.

### Approach During Surgical Cricothyrotomy

When performing surgical CTTs, dissection through the soft tissue and incision into the CTM could be considered of more importance than percutaneous kits, as tissue dilators are not used. In order to achieve an adequate incision length, one study suggested a single stab incision through the skin and CTM at a 60° angle with a #20 blade could produce an incision of approximately 2.5 cm (18). This should be more than sufficient to accommodate any tube that may be used for cricothyrotomy. We confirmed this concept using a #22 blade for the surgical technique in cadaver dogs. Unfortunately, kits such as those tested in this study come with #10 and #15 blades. Therefore, if using these smaller blades, a conscious effort must be made to incise the overlying muscle and the CTM to a greater length than produced with a stab incision alone to allow passage of a tube.

Furthermore, the authors recommend that after soft tissue dissection, the entire sagittal length of the CTM may be incised from thyroid to cricoid cartilage to prevent failure of tube passage. If there is doubt the incision is sufficient, one report suggested that the operator's little finger be used as a guide for incision length; If the incision can accommodate the tip of the operator's little finger to the depth of the airway, a minimum of a 6.0 mm I.D. endotracheal tube can be used (20).

### Emergency Awake CTT

The novel awake cricothyrotomy has two main advantages: (1) reduced anaesthetic requirement (2) sternal recumbent position.

Anaesthetic induction agents typically reduce respiratory drive and can lower blood pressure. This is not ideal in an animal that already has respiratory compromise and likely severely hypoxaemic. Furthermore, circulation may be compromised if trauma is the necessitating indication of eFONA.

This technique positions the dog in sternal recumbency (prone), which allows more effective ventilation and perfusion matching than dorsal recumbency (supine) (32). Further, if switching from a failed intubation in sternal recumbency, maintaining the dog in this position reduces repositioning time and allows for continued optimized matching of ventilation and perfusion. It should be noted that animals in respiratory distress, requiring awake CTT, may be orthopneic in a sitting or standing position. In these truly emergent situations, we would not recommend forcing the patient into sternal recumbency as the site may still be accessed by dorsiflexion of the neck without changing the animal's position.

Rotating the scalpel blade proximally is presented as a further adaptation to the surgical cricothyrotomy in the dog to avoid a complication known as bougie trapping. Previous reports demonstrated that bevelled incisions were made into a gum elastic bougie while repositioning it in the airway and leaving the scalpel *in situ* for tissue retraction. This can create a flap on the bougie surface that, during withdrawal of the bougie, catches onto the end of the CTT tube resulting inadvertent tube removal (33). The step of rotating the scalpel blade in a proximal direction, the sternal recumbent position and recommendations for the procedure to be performed without general anaesthesia has not previously been described or discussed in the veterinary literature.

### Benefits of CTT Over Other Airway Techniques

The CTT provides several advantages over the other advanced airway management techniques. Unlike orotracheal intubation, the CTT bypasses the glottis and, therefore, does not require general anaesthesia to maintain the tube *in situ*. Invasive management of the airway in the conscious patient has the benefit of reducing the amount of anaesthetic medication required, which may be detrimental to the animal's cardiovascular and respiratory systems, particularly after suffering trauma.

Cricothyrotomies can accommodate large diameter tubes, thereby reducing the work of breathing. One report recommends tube sizes with internal diameters (I.D.) from 9 to 11 mm for TTs in dogs (2). In the author's experience, it is a struggle to even insert tubes larger than 5 mm I.D. in to tracheostomy incisions, even in large dogs. However, all the dogs in this report easily accommodated a 10.0 mm I.D., cuffed, ETT tube in the cricothyroid space, which was placed as part of the accompanying study (Part 2), assessing ventilation through cricothyrotomy tubes.

When compared with TT, CTT is the faster technique when performed by novices (12). Due to its relative simplicity, CTT is likely a more readily retainable skill that may be more easily applied by HCPs with limited surgical training or those not performing advanced airway techniques regularly.

The CTT is already the preferred and, nearly, exclusively practised emergent surgical out-of-hospital airway technique. Military and civilian prehospital providers alike are already predominantly trained and experienced in performing CTTs; few, if any, are trained in TT. This makes the CTT directly translatable to most HCP's current knowledge, experience, and competency.

Finally, complications of TT when compared to CTT include a greater chance of damaging blood vessels and delayed major vessel haemorrhage, particularly if performed during low blood pressure states (34).

### Limitations

The authors recognize the following limitations to the study. It is possible that freezing and thawing or refrigeration may have led to alteration of the tissue characteristics in our cadavers. Application of the CTT kits using the same steps for humans may still be possible in live tissue. Furthermore, the two kits that required tissue dilation (PCK and Melker) function on the same principles and similar results should be expected, however the Melker required more dissection. Although unlikely, tissue characteristics of individual cadavers in our small sample size may have led to the different success or failure rates for each kit. Overall, the Melker kit was placed in three cadavers and the PCK in two. Surgical techniques were performed for the remaining cadavers.

Cricothyrotomy kits are expensive (Melker universal: $937.49 USD, Portex®: $245.50 USD and H&H® $57.99 USD), hence, may not be relevant to primary veterinary care providers due to the cost and infrequency of use. Note: The Melker kit can be purchased as a Seldinger kit alone at $225.00 USD. Furthermore, all equipment for a surgical technique is readily available in veterinary hospitals, with the added benefit of being able to choose the most appropriate tube size.

### Conclusion

The author's experiences with the PCK, Melker and H&H® emergency cricothyrotomy kits have been described. This is the first publication to include detailed step-by-step instruction with images and video of the application of emergency cricothyrotomy kits and a novel surgical technique in cadaver dogs. This paper can be used as a basic framework to guide veterinarians in performing the CTT procedure and pre-hospital HCP in performing emergency CTT in working dogs using various techniques.

## Data Availability Statement

The raw data supporting the conclusions of this article will be made available by the authors, without undue reservation.

## Ethics Statement

The animal study was reviewed and approved by The University of Queensland Production and Companion Animal Ethics Committee, Certificate No. ANRFA/159/20.

## Author Contributions

SH conceived the experiment, acquired the equipment, performed the methodology, and authored the initial draft of the manuscript. MH contributed to the performing the methodology. MH and LP contributed to authorship and revisions of the manuscript. All authors contributed to the article and approved the submitted version.

## Funding

Funding for the acquisition and use of equipment was provided by the University of Queensland Research Donation Fund (Grant No. 2019002893), whose role is to provide funding to improve health and welfare of companion animals. Funding for open access publication fees were provided by MH.

## Conflict of Interest

The Portex PCK was unconditionally donated by Smiths Medical Australia. The authors declare that the research was conducted in the absence of any commercial or financial relationships that could be construed as a potential conflict of interest.

## Publisher's Note

All claims expressed in this article are solely those of the authors and do not necessarily represent those of their affiliated organizations, or those of the publisher, the editors and the reviewers. Any product that may be evaluated in this article, or claim that may be made by its manufacturer, is not guaranteed or endorsed by the publisher.

## Footnotes

^1^Smiths Medical Australasia Macquarie Park, NSW 2113, Australia.

^2^Cook Medical LLC, Bloomington, IN 47402, USA.

^3^H&H medical corporation, Williamsburg, VA 23185, USA.

^4^ https://www.youtube.com/watch?v=IzDh4WSjmNU

^5^ https://youtu.be/J3FIbmMi-lA

^6^ https://youtu.be/PHAGfJYsAxo

^7^ https://youtu.be/XCI429J6hs0
