# Supplementary figures and images for: Prehospital Emergency Cricothyrotomy in Dogs Part 1: Experiences With Commercial Cricothyrotomy Kits
Source: Front Vet Sci. 2021 Sep 16;8:705695. doi: 10.3389/fvets.2021.705695 (PMC8483268; doi:10.3389/fvets.2021.705695)

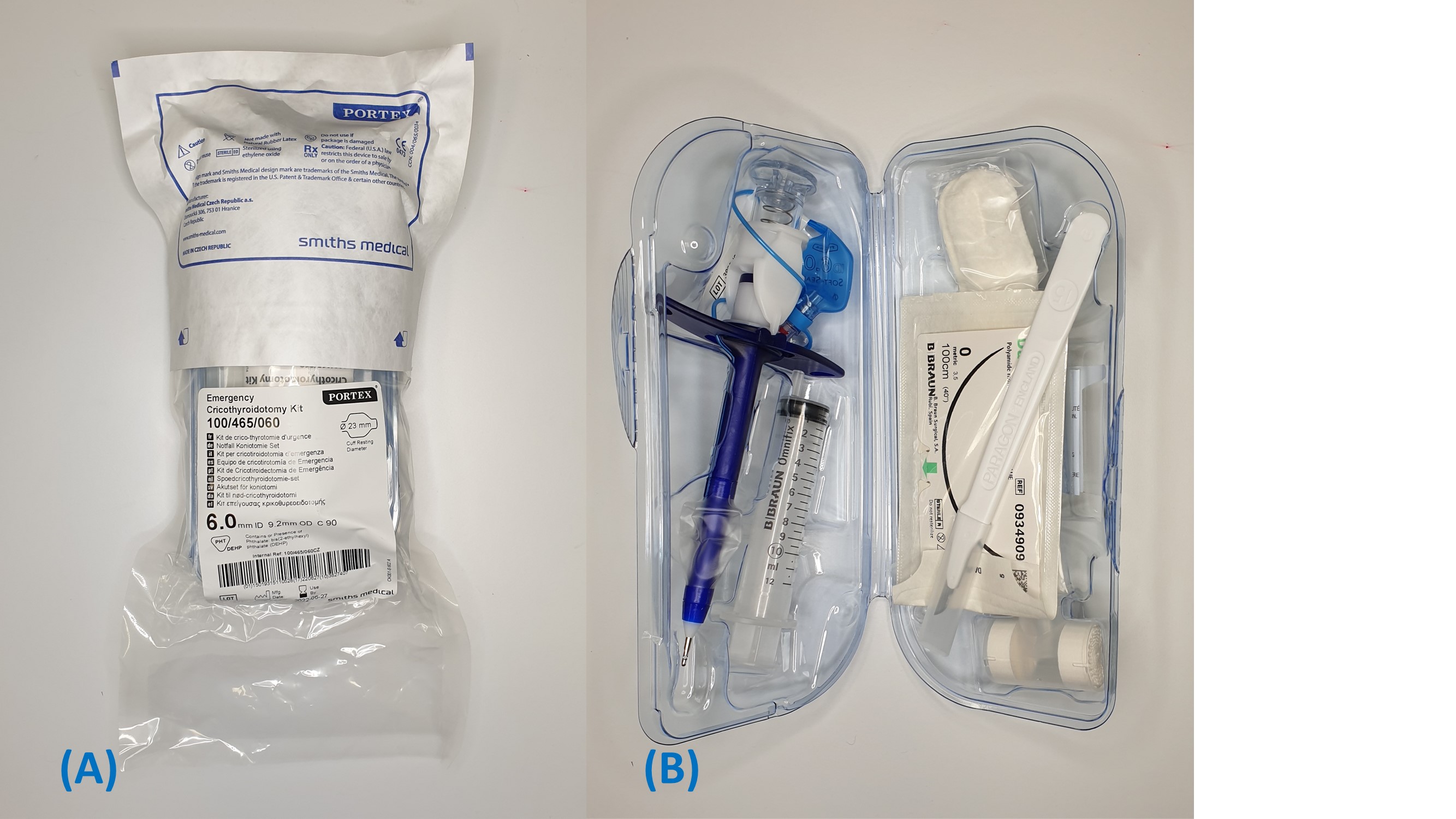

Supplement: Supplementary file 1 [file Data_Sheet_1.ZIP › 2.1 A-B.JPG]

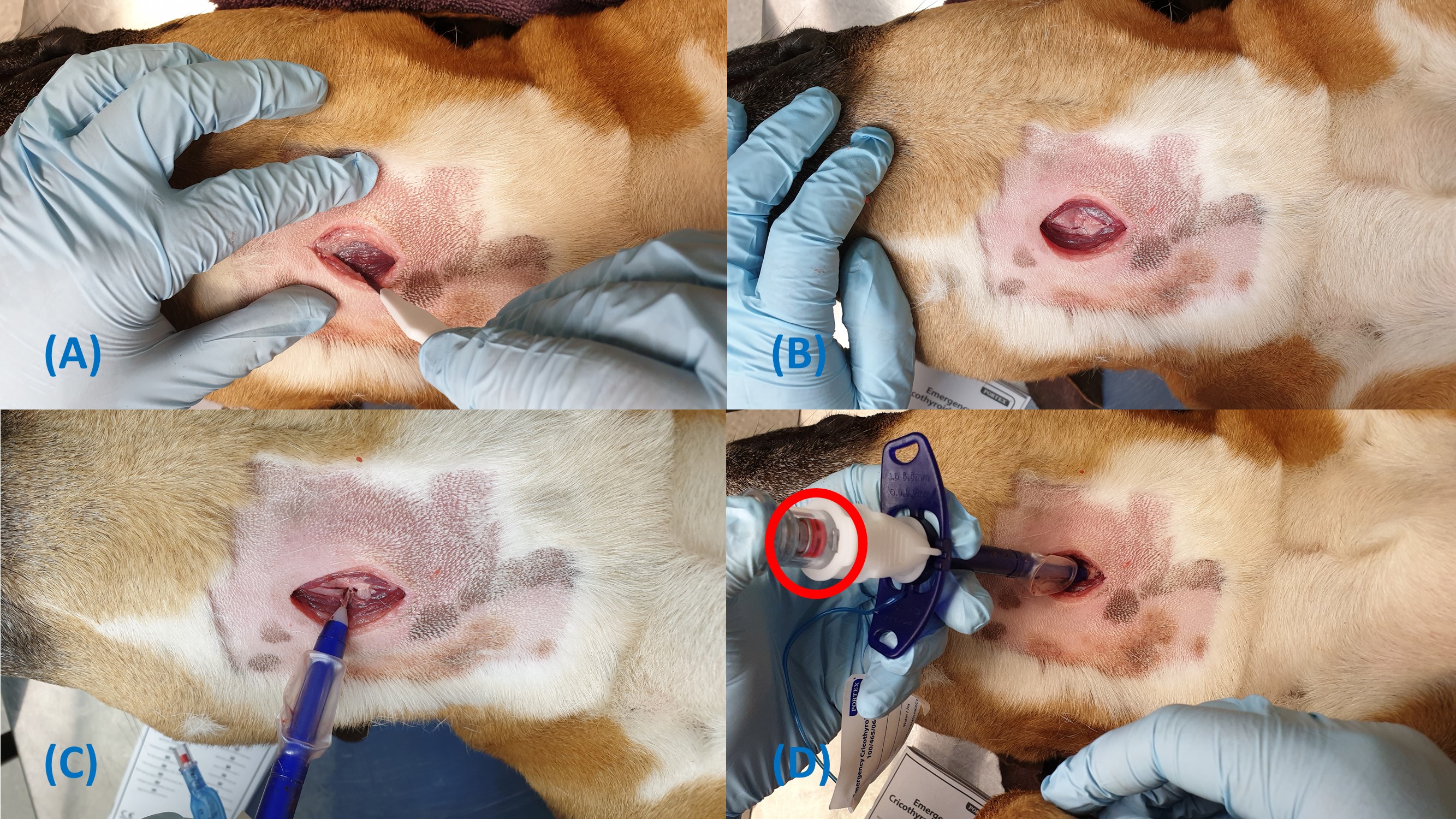

Supplement: Supplementary file 1 [file Data_Sheet_1.ZIP › 2.2 - A-D .JPG]

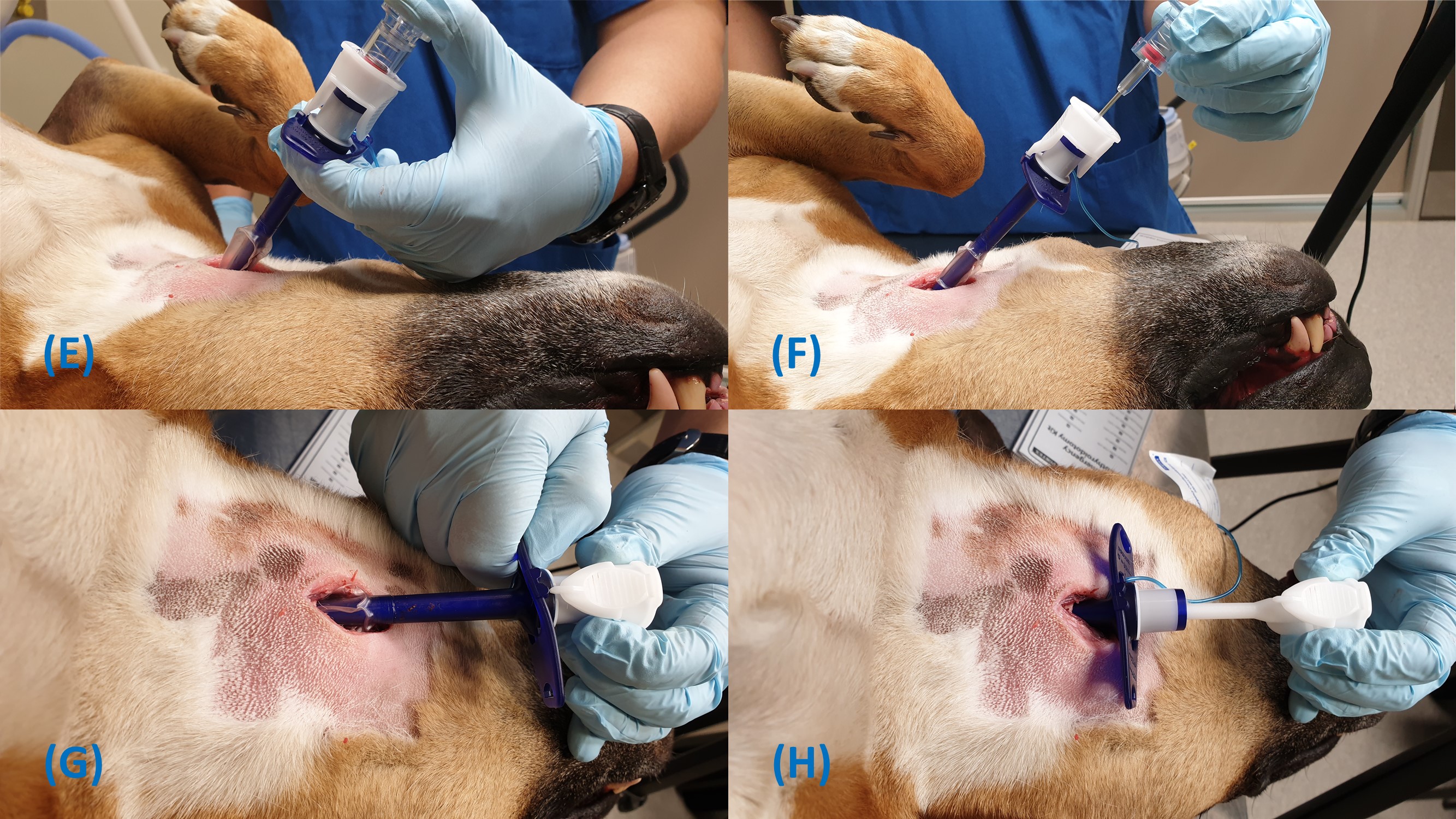

Supplement: Supplementary file 1 [file Data_Sheet_1.ZIP › 2.2 - E-H .JPG]

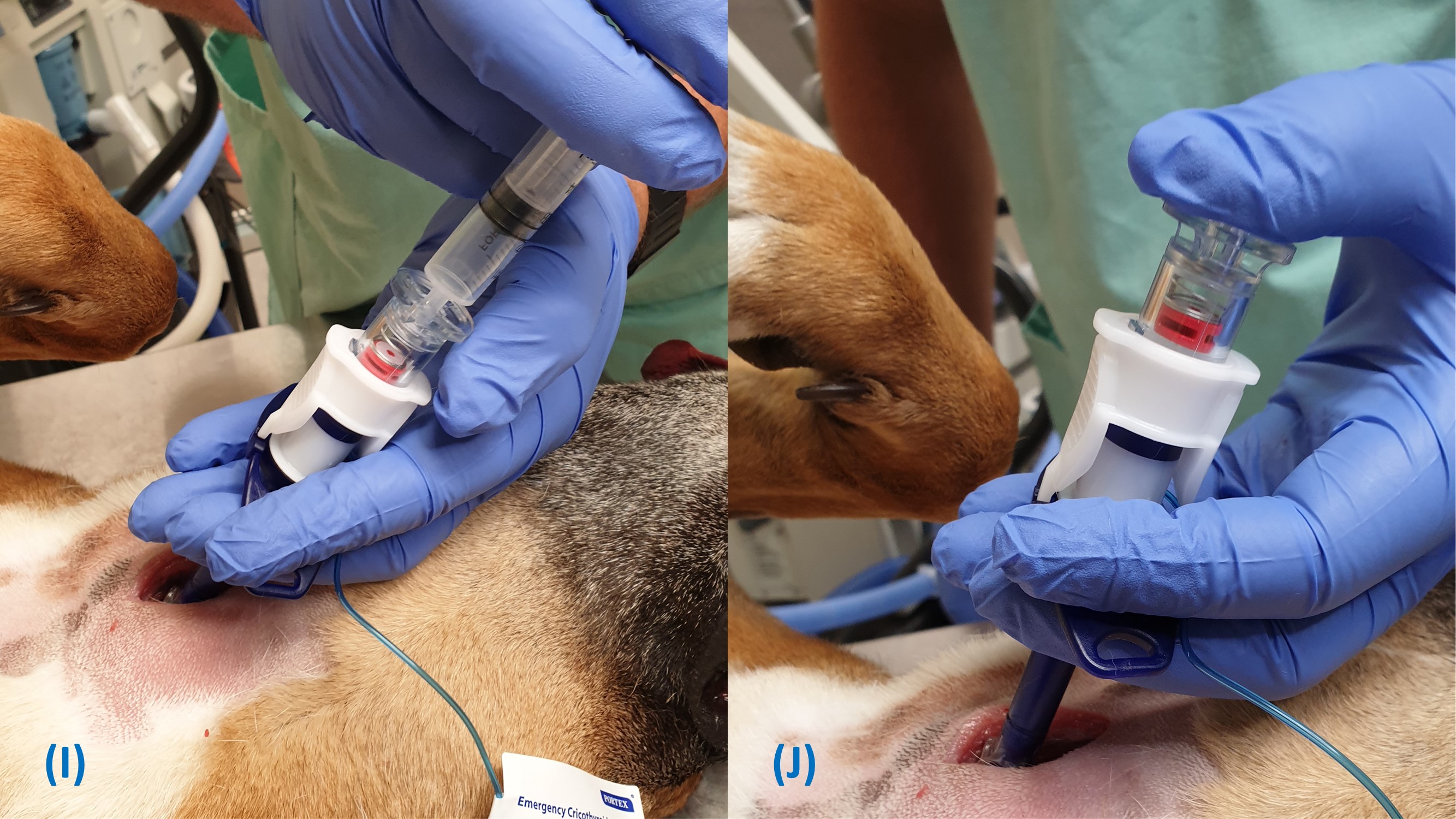

Supplement: Supplementary file 1 [file Data_Sheet_1.ZIP › 2.2 I-J .JPG]

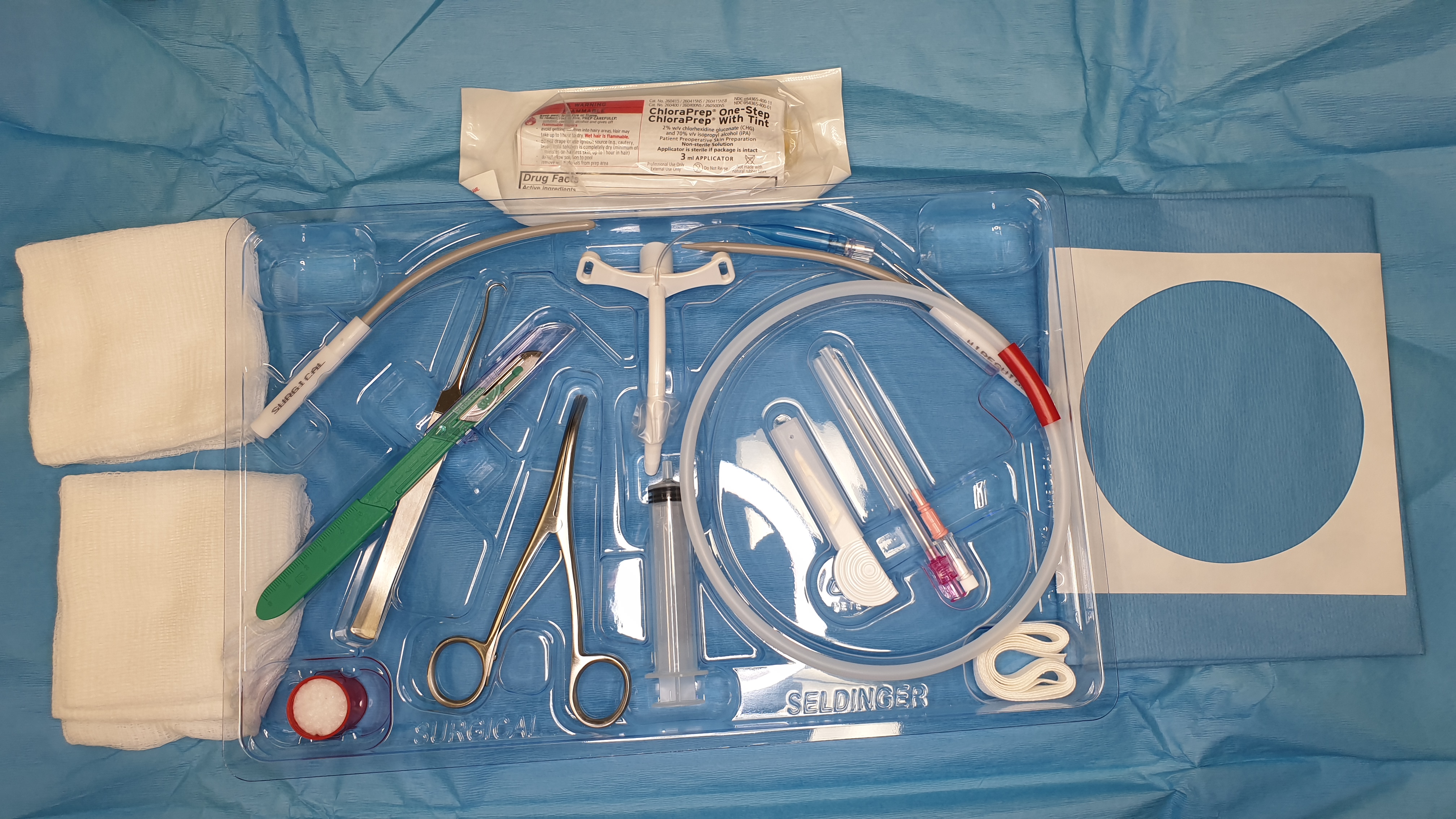

Supplement: Supplementary file 1 [file Data_Sheet_1.ZIP › 3.1.jpg]

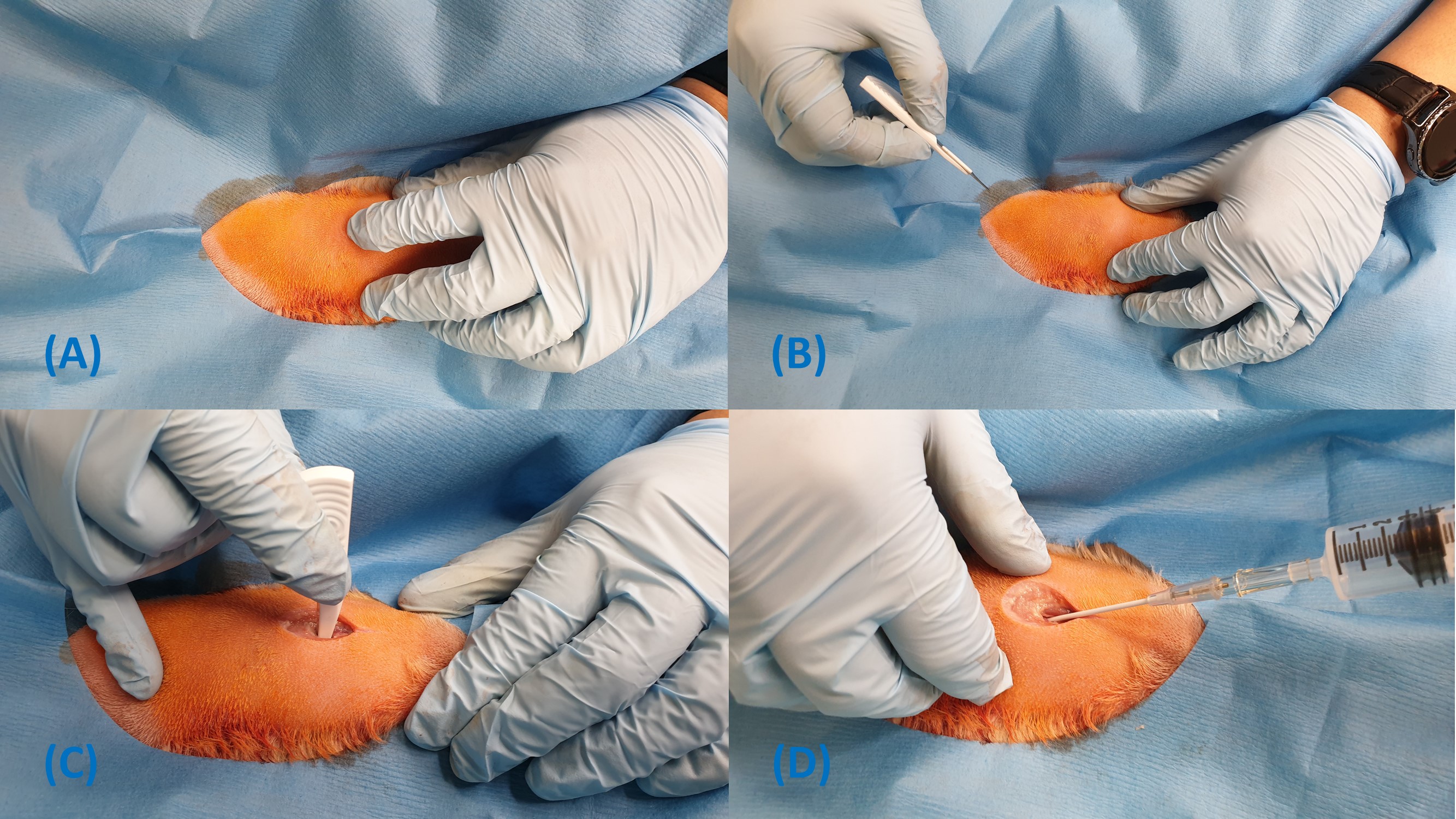

Supplement: Supplementary file 1 [file Data_Sheet_1.ZIP › 3.2 A-D .JPG]

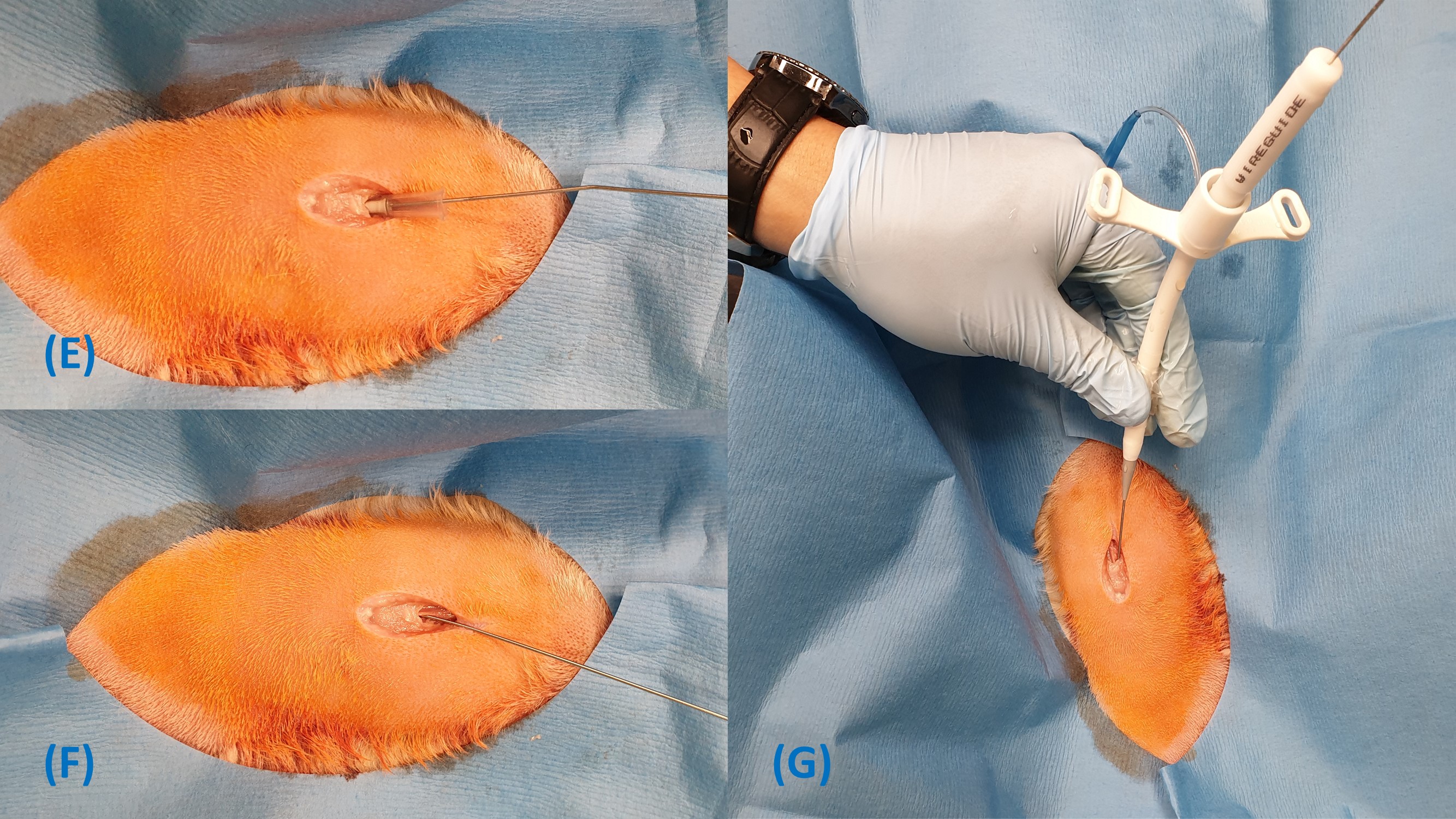

Supplement: Supplementary file 1 [file Data_Sheet_1.ZIP › 3.2 E-G .JPG]

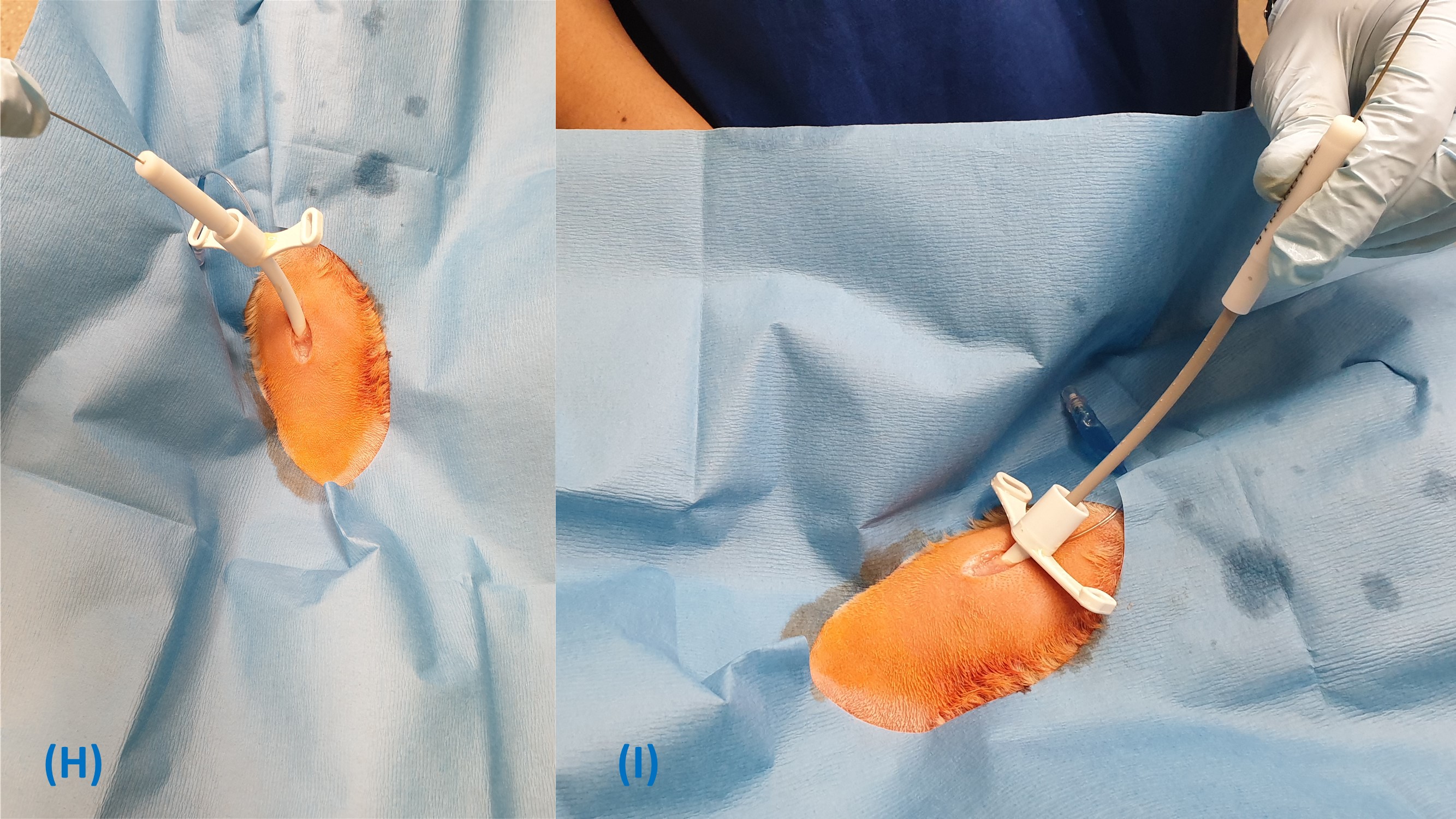

Supplement: Supplementary file 1 [file Data_Sheet_1.ZIP › 3.2 H-I .JPG]

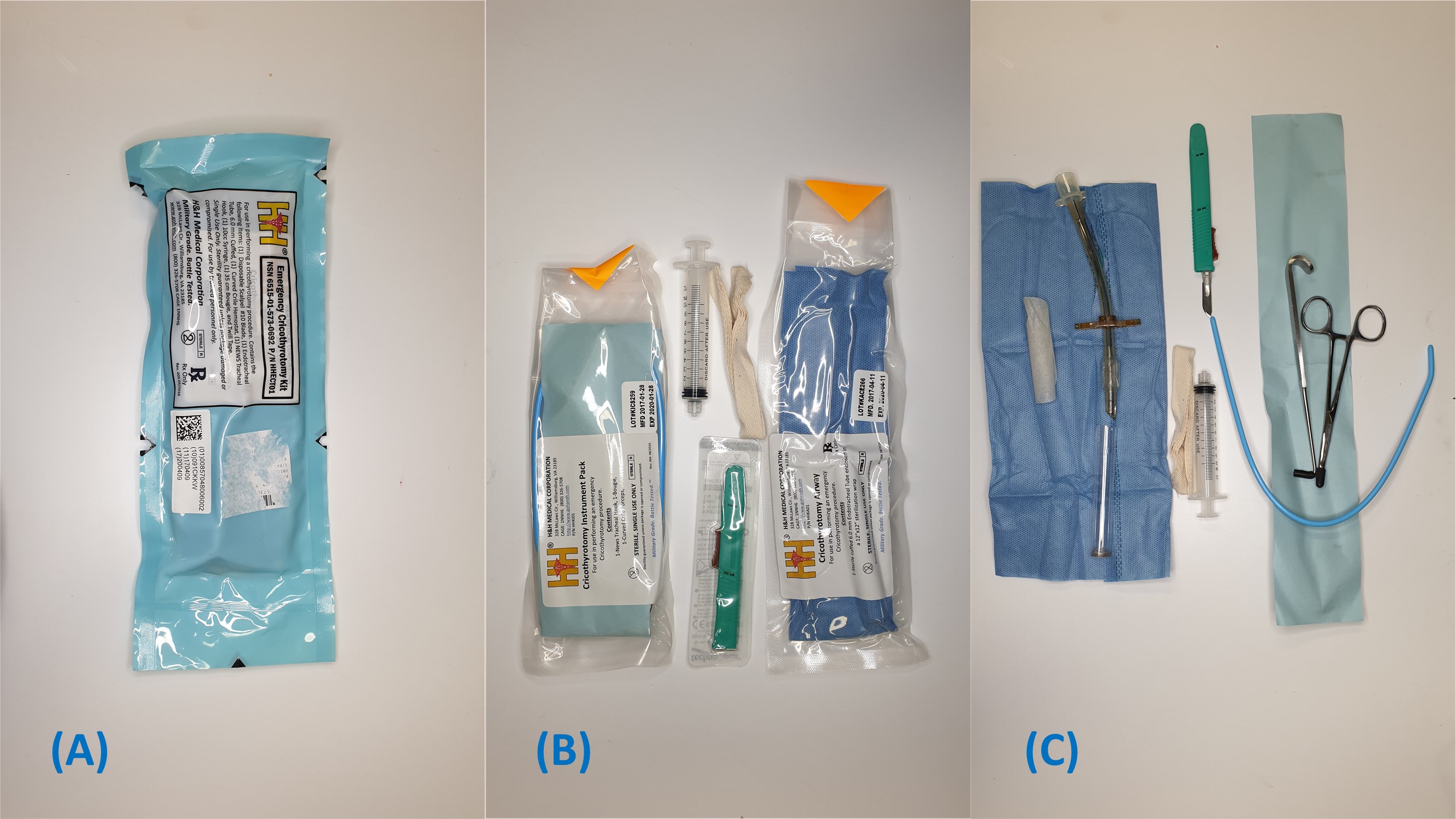

Supplement: Supplementary file 1 [file Data_Sheet_1.ZIP › 4.1 A-C .JPG]

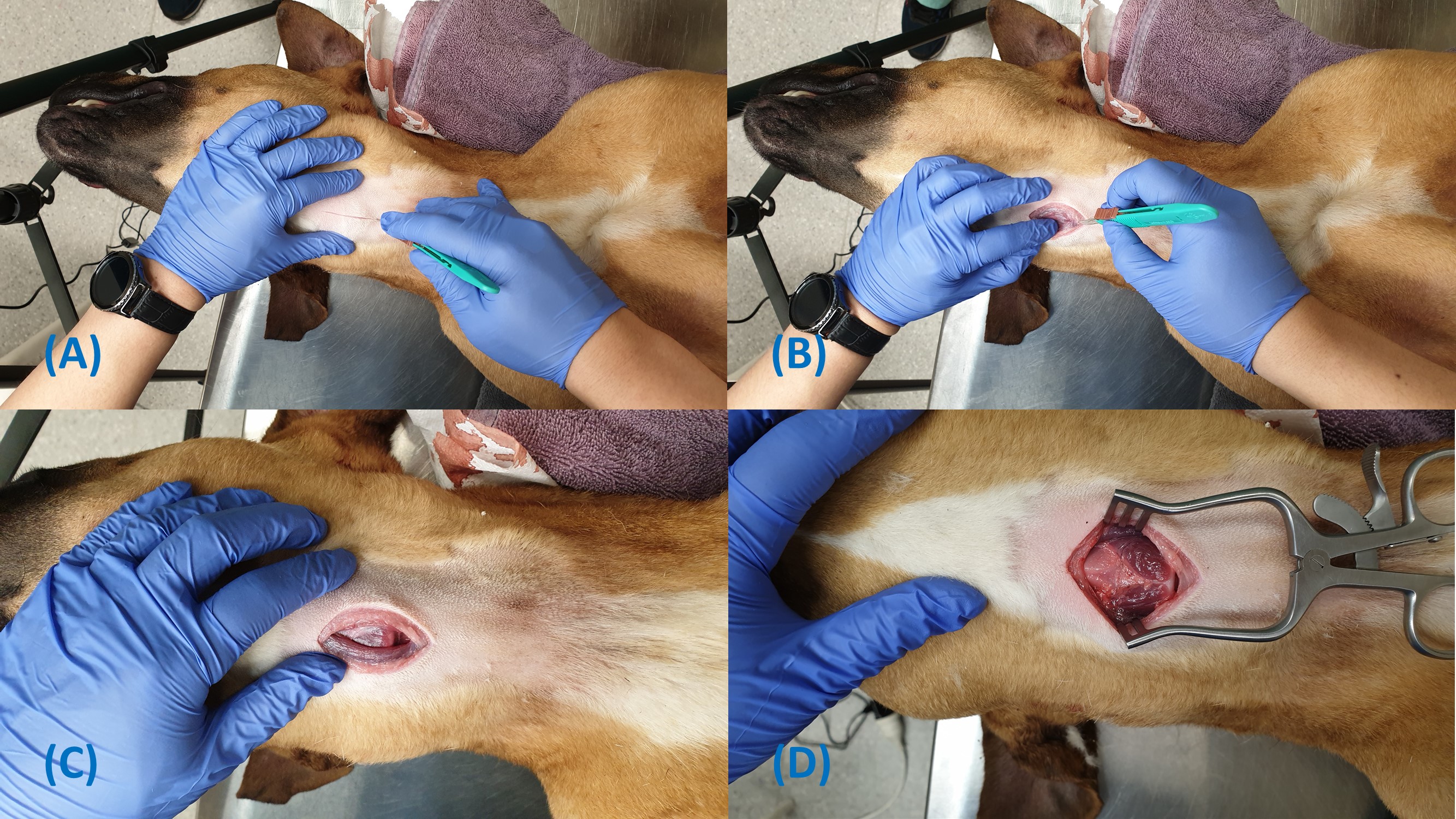

Supplement: Supplementary file 1 [file Data_Sheet_1.ZIP › 4.2 A-D .JPG]

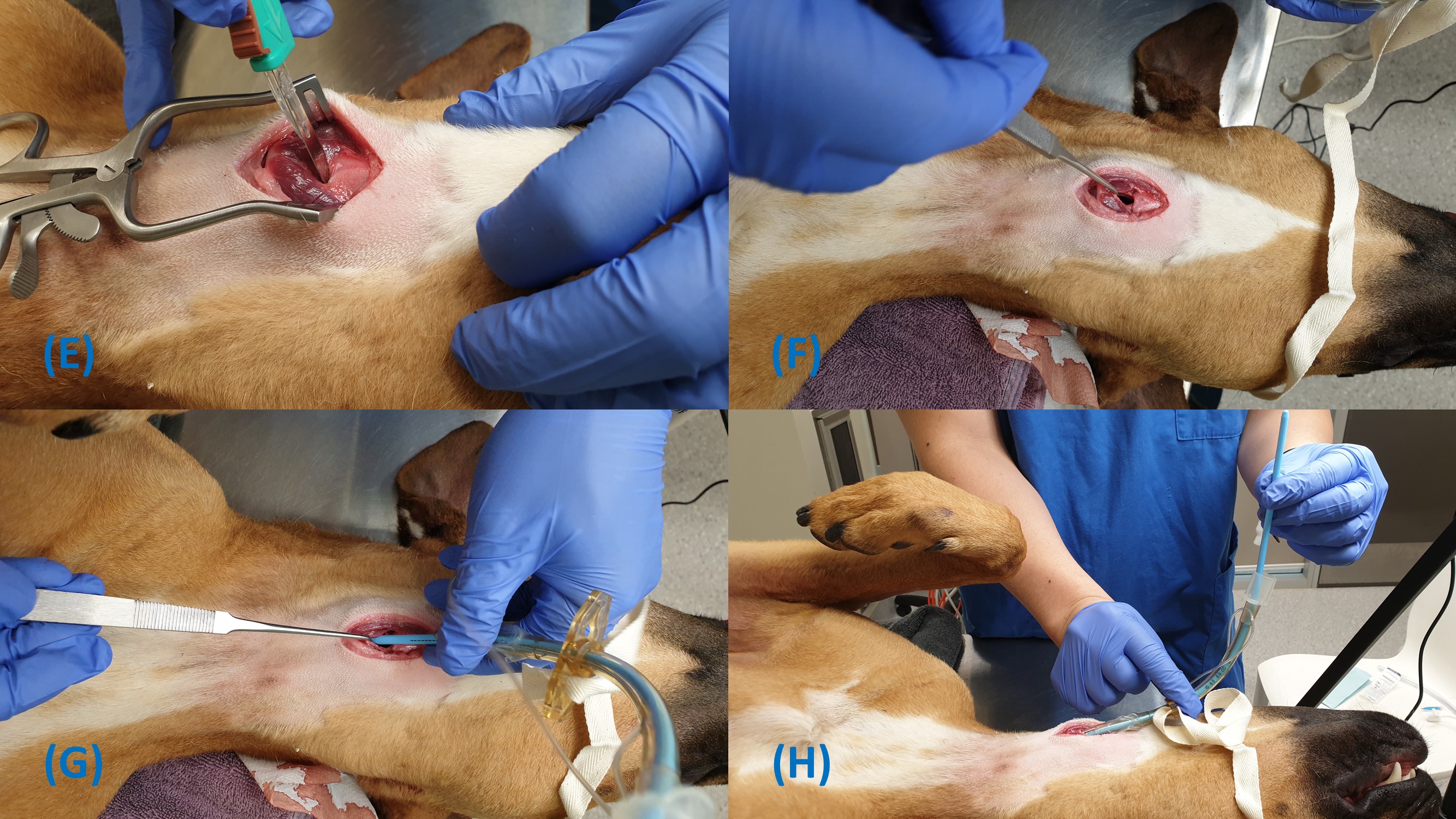

Supplement: Supplementary file 1 [file Data_Sheet_1.ZIP › 4.2 E-H .JPG]

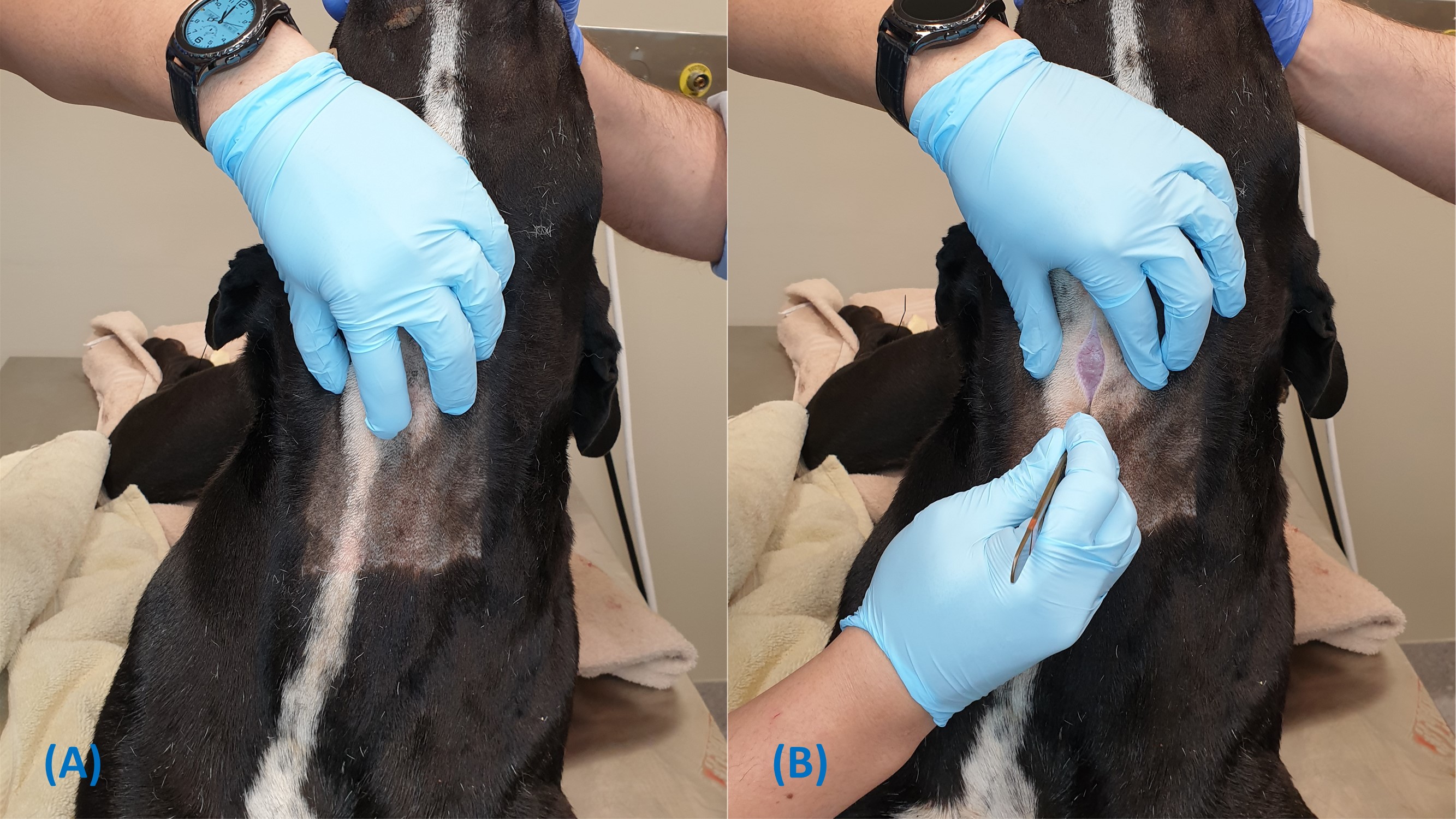

Supplement: Supplementary file 1 [file Data_Sheet_1.ZIP › 5 A-B .JPG]

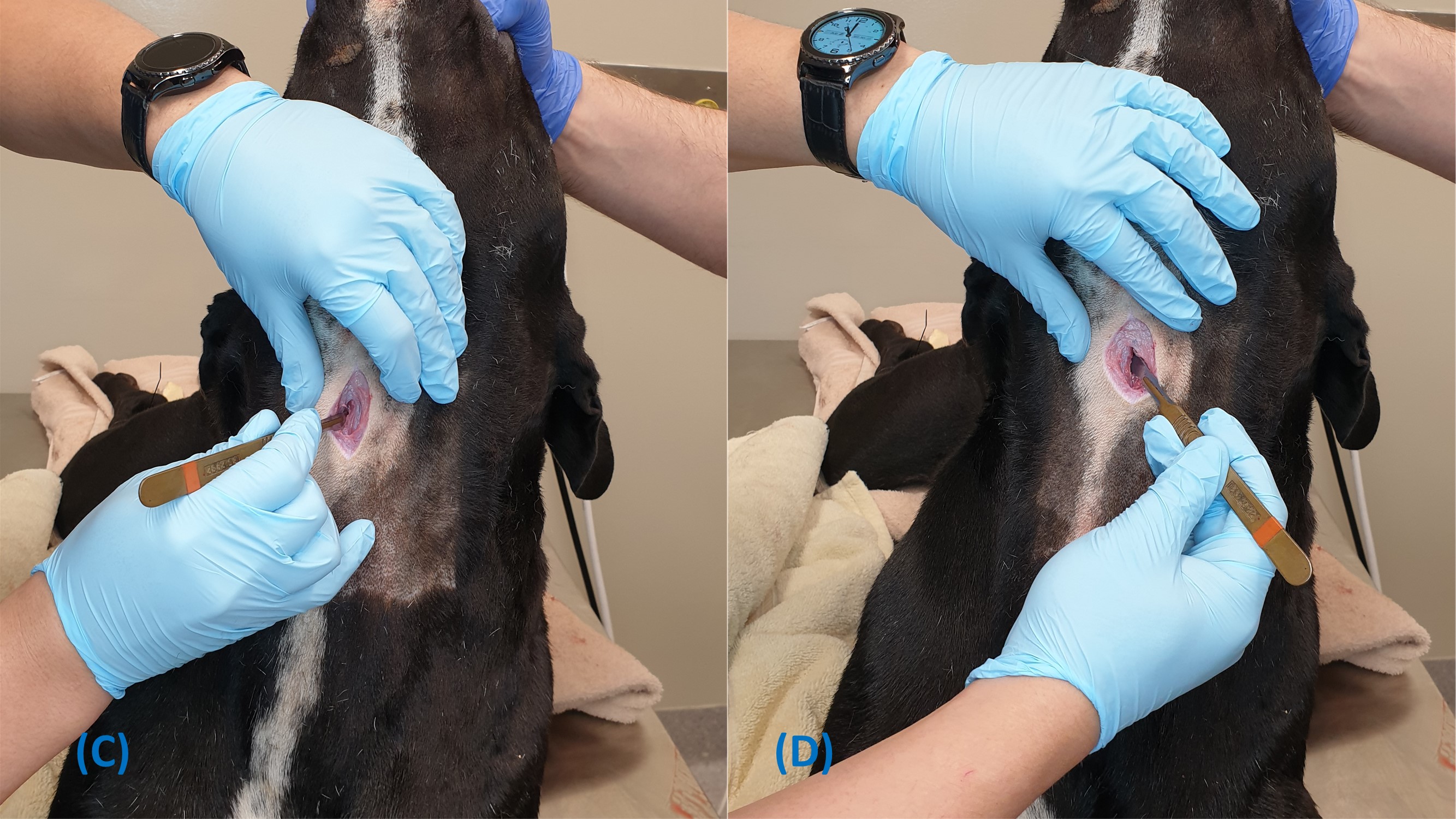

Supplement: Supplementary file 1 [file Data_Sheet_1.ZIP › 5 C-D.JPG]

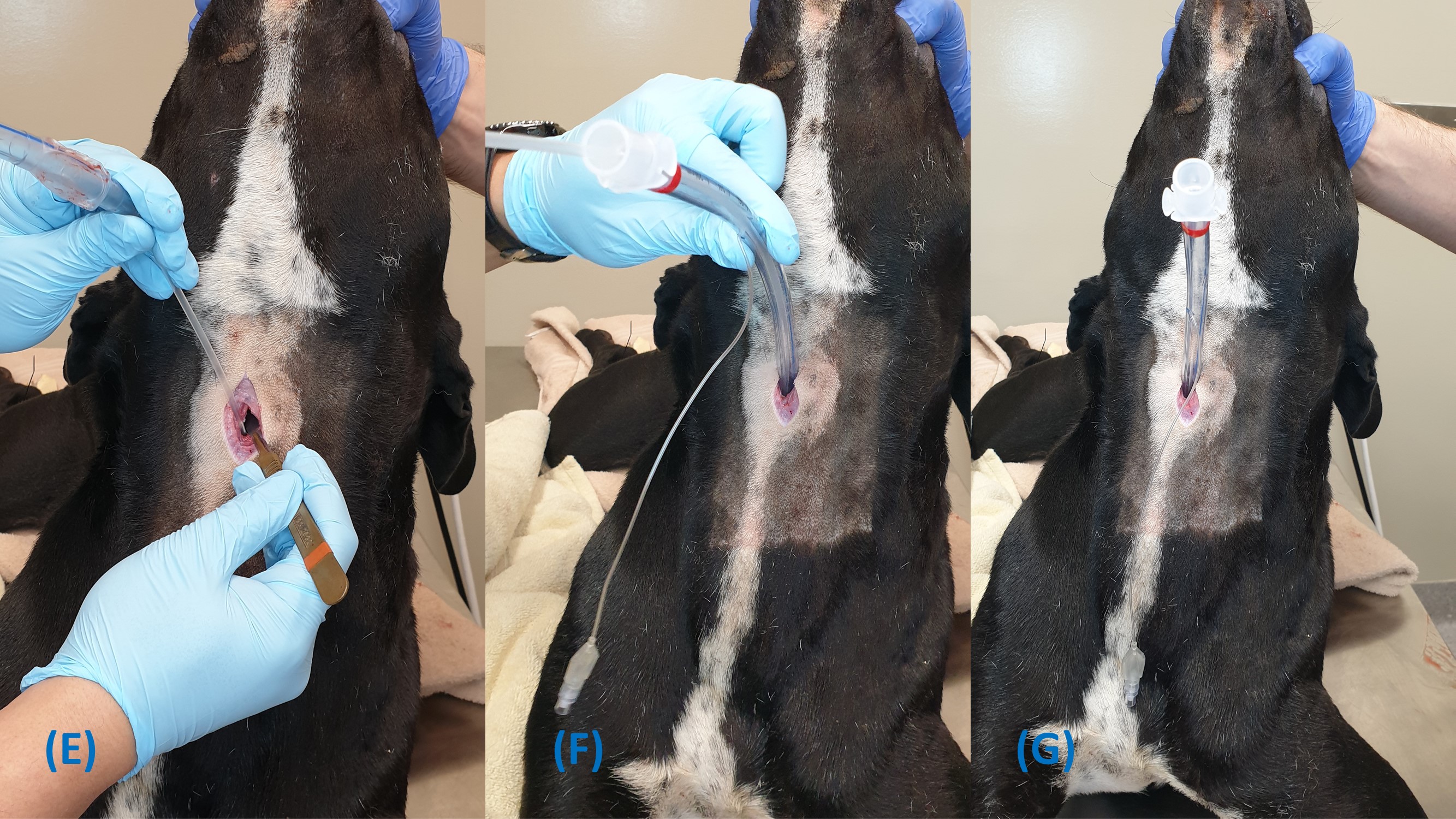

Supplement: Supplementary file 1 [file Data_Sheet_1.ZIP › 5 E-G .JPG]

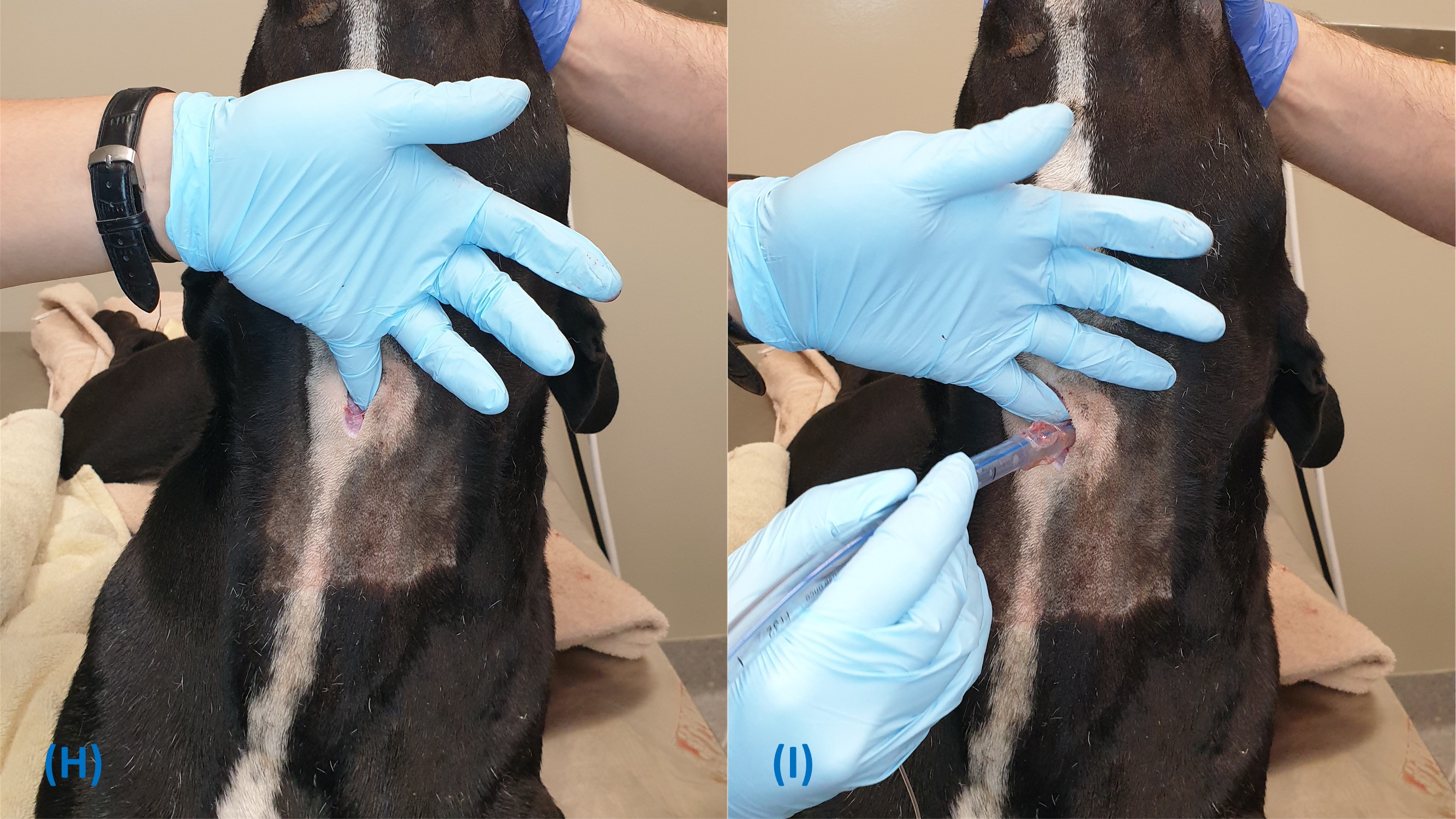

Supplement: Supplementary file 1 [file Data_Sheet_1.ZIP › 5 H-I .JPG]

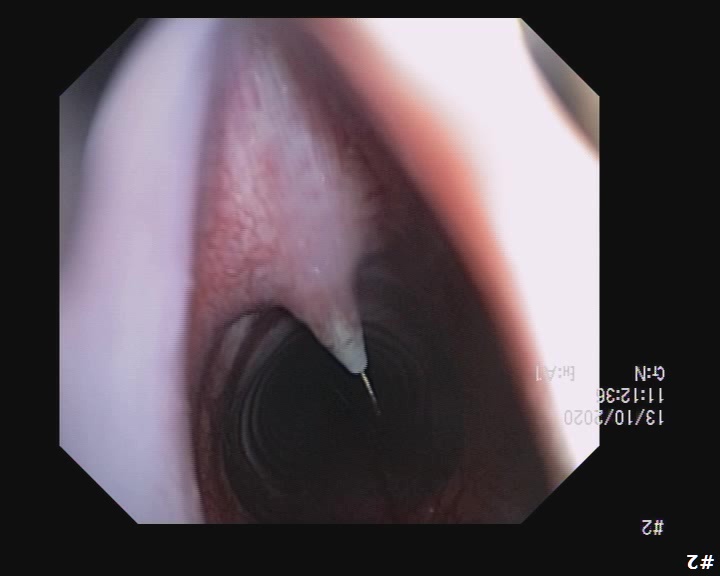

Supplement: Supplementary file 1 [file Data_Sheet_1.ZIP › 6 A.jpg]

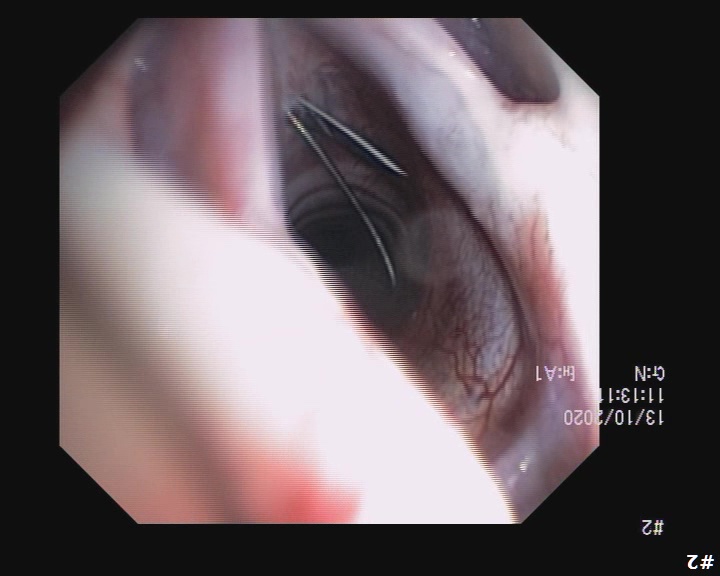

Supplement: Supplementary file 1 [file Data_Sheet_1.ZIP › 6 B.jpg]

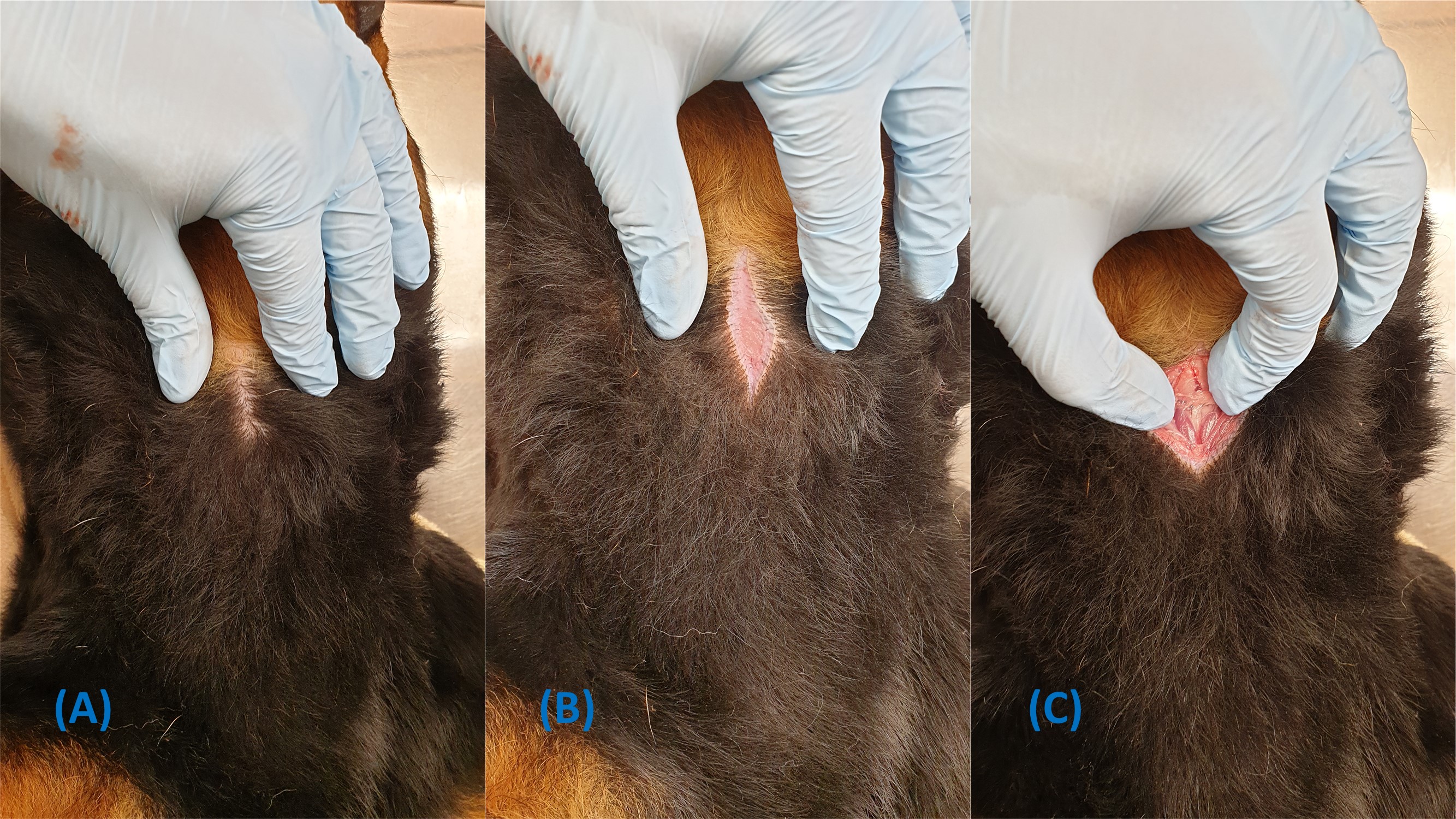

Supplement: Supplementary file 1 [file Data_Sheet_1.ZIP › 1 - A-C .JPG]

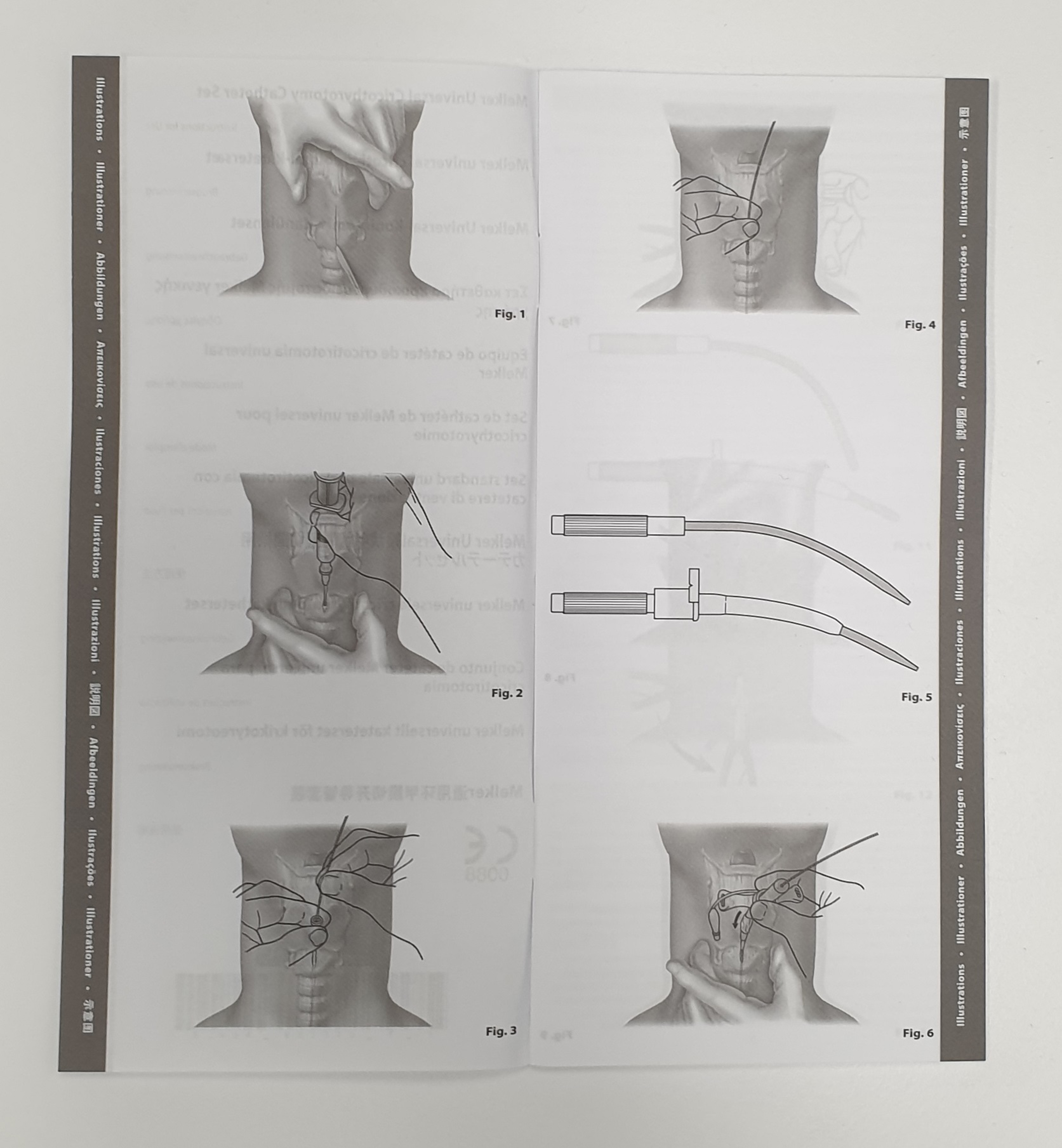

Supplement: Supplementary file 2 [file Image_1.JPEG]

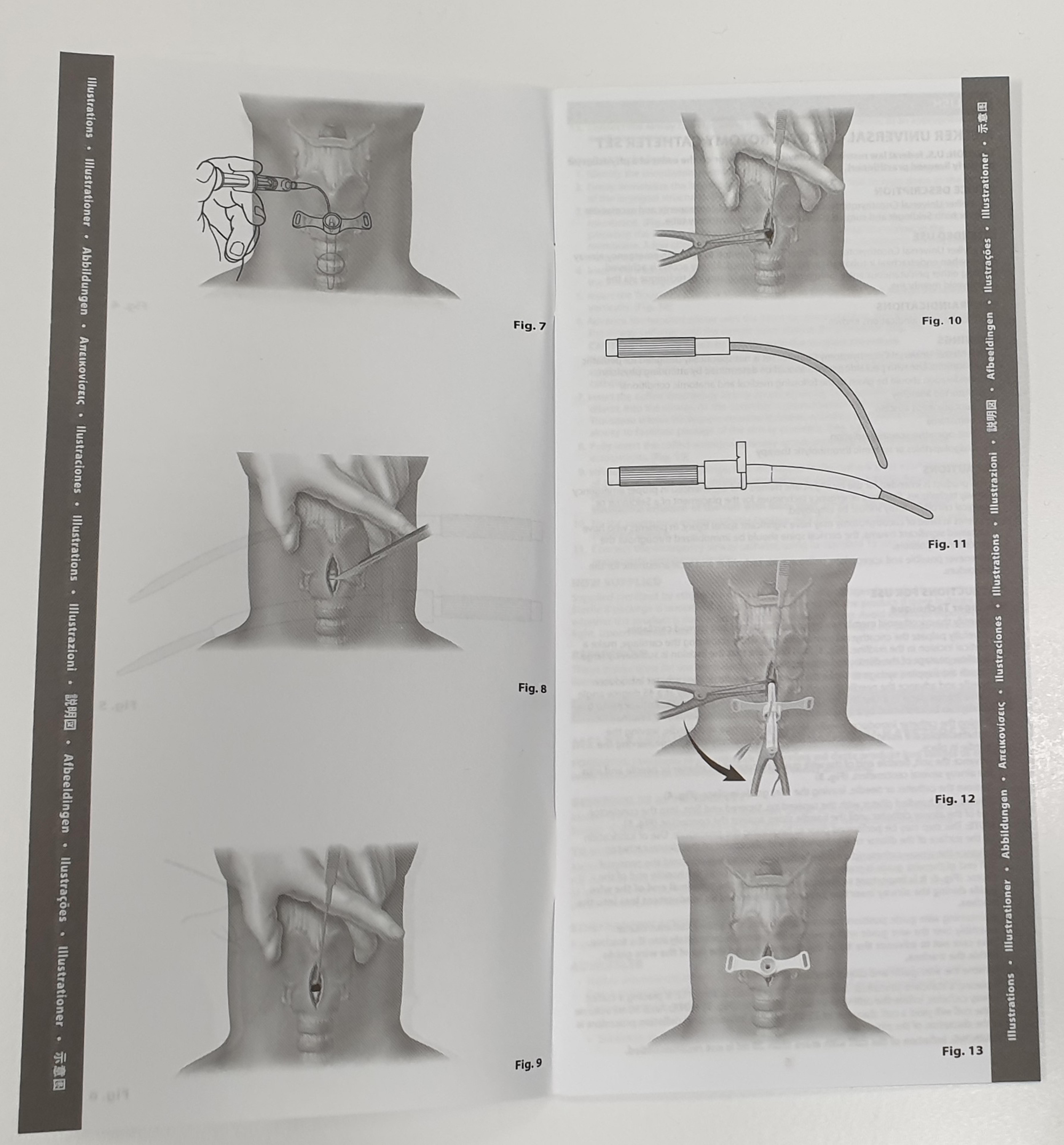

Supplement: Supplementary file 3 [file Image_2.JPEG]

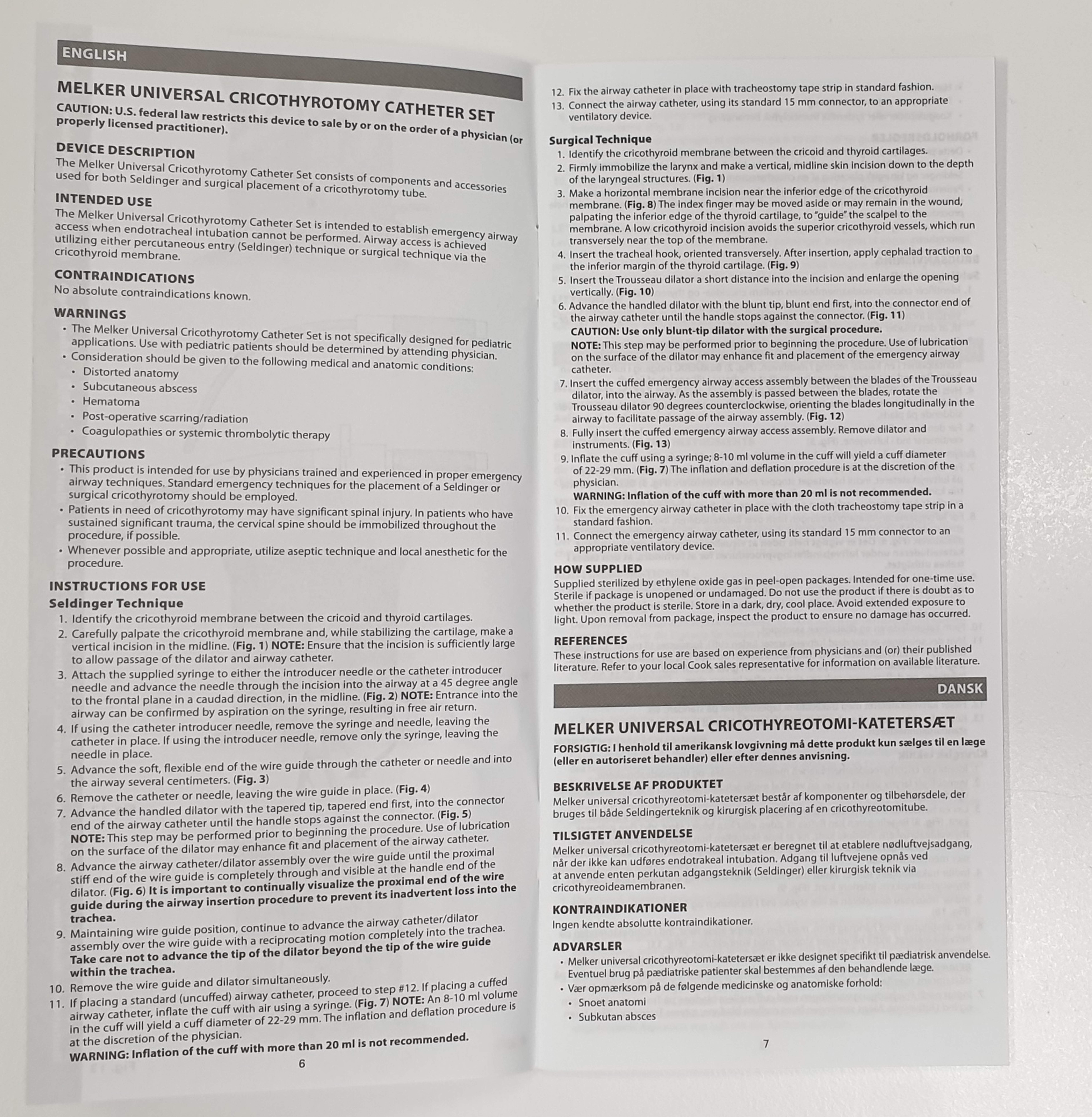

Supplement: Supplementary file 4 [file Image_3.JPEG]

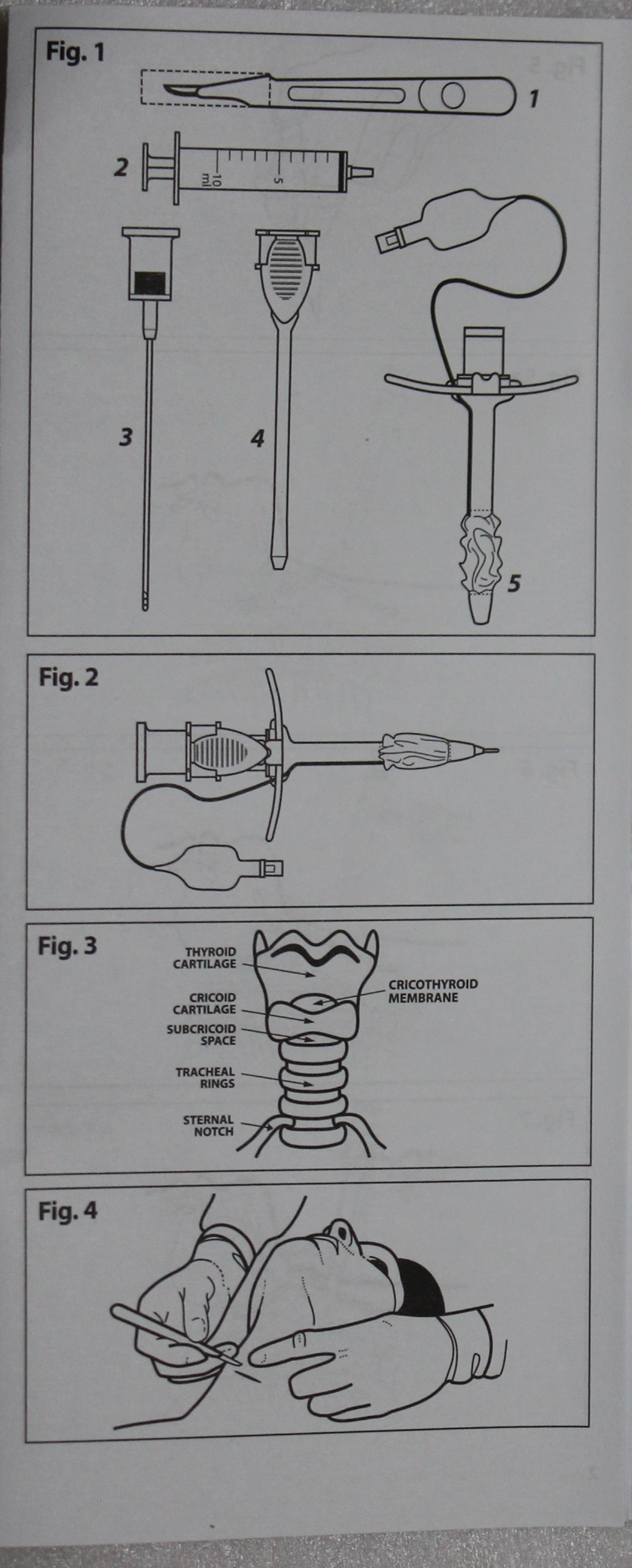

Supplement: Supplementary file 5 [file Image_4.JPEG]

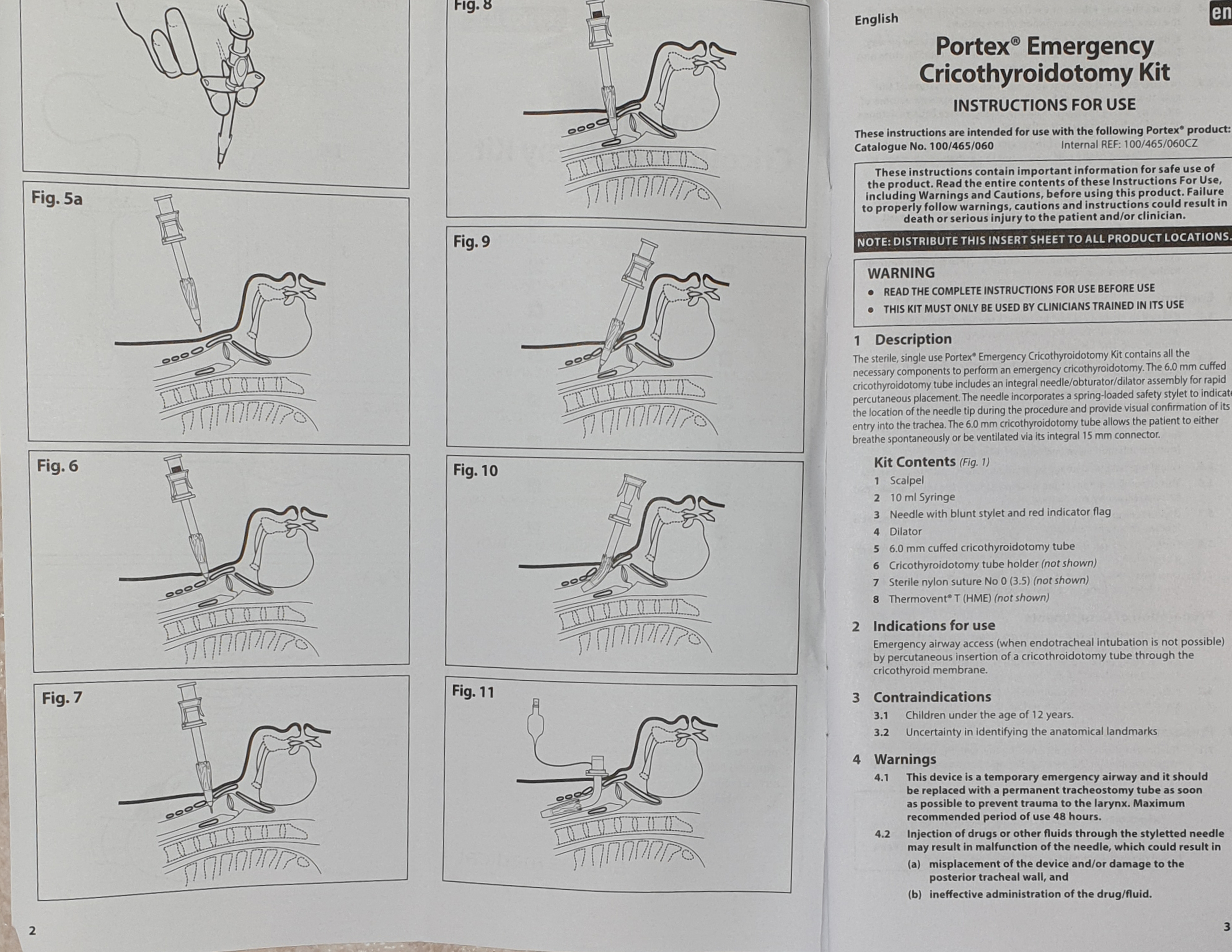

Supplement: Supplementary file 6 [file Image_5.JPEG]

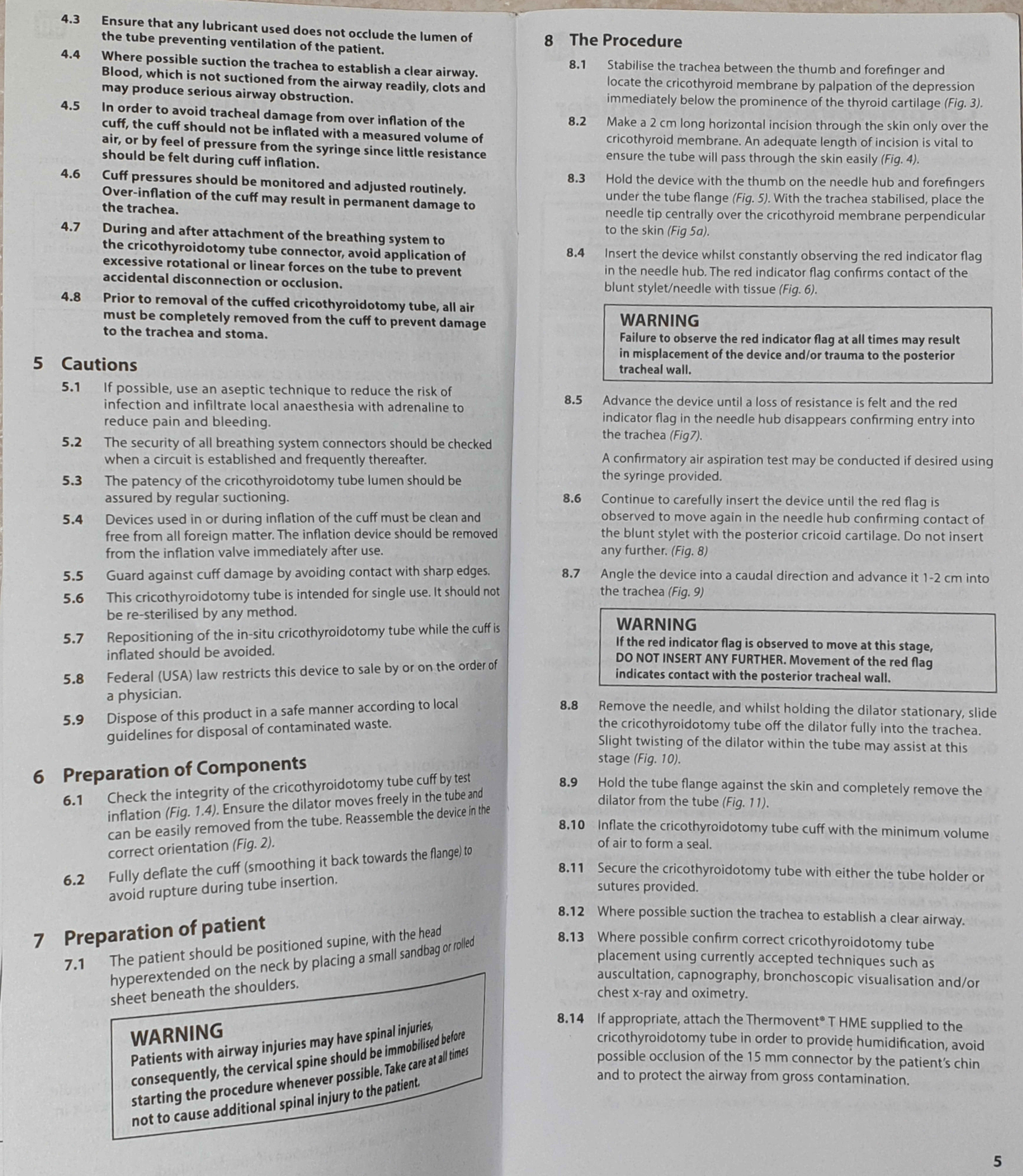

Supplement: Supplementary file 7 [file Image_6.JPEG]
